# Predicting Accurate Lead Structures for Screening Molecular Libraries: A Quantum Crystallographic Approach

**DOI:** 10.3390/molecules26092605

**Published:** 2021-04-29

**Authors:** Suman Kumar Mandal, Parthapratim Munshi

**Affiliations:** Chemical and Biological Crystallography, Department of Chemistry, School of Natural Sciences, Shiv Nadar University, Dadri 201314, Uttar Pradesh, India; sk225@snu.edu.in

**Keywords:** lead structure, molecular docking, scoring function, kernel energy method, quantum crystallography, protein-ligand interaction

## Abstract

Optimization of lead structures is crucial for drug discovery. However, the accuracy of such a prediction using the traditional molecular docking approach remains a major concern. Our study demonstrates that the employment of quantum crystallographic approach-counterpoise corrected kernel energy method (KEM-CP) can improve the accuracy by and large. We select human aldose reductase at 0.66 Å, cyclin dependent kinase 2 at 2.0 Å and estrogen receptor β at 2.7 Å resolutions with active site environment ranging from highly hydrophilic to moderate to highly hydrophobic and several of their known ligands. Overall, the use of KEM-CP alongside the GoldScore resulted superior prediction than the GoldScore alone. Unlike GoldScore, the KEM-CP approach is neither environment-specific nor structural resolution dependent, which highlights its versatility. Further, the ranking of the ligands based on the KEM-CP results correlated well with that of the experimental IC_50_ values. This computationally inexpensive yet simple approach is expected to ease the process of virtual screening of potent ligands, and it would advance the drug discovery research.

## 1. Introduction

Lead optimization is an essential part of drug discovery, where a weakly potent substrate/lead structure, identified by virtual or high throughput screening, is developed by improving ligand specificity, potency, and pharmacokinetic properties. One of the efficient ways of accelerating the lead optimization process is to predict the ligand binding affinity and/or functional potency, as it cuts down the labour and reduces the cost. Various methods have been developed and reviewed for calculating ligand binding affinity [1,2]. Methods such as molecular dynamic simulations, free energy perturbation, Monte Carlo simulations and thermodynamic integration can calculate binding free energies that are comparable to the experimentally determined values [3,4,5]. Molecular Mechanics/Poisson Boltzmann Surface Area (MM/PBSA) calculations compute binding free energies between the bound and the unbound states for the binding complexes using a combination of MM and continuum solvation [6]. A relatively similar approach MM/Generalized Born Surface Area (GBSA) has been used to study protein-ligand interactions and is applied to diverse targets [7,8]. Although free energy calculations using the aforementioned methods have produced promising results to some extent [9], these approaches are computationally expensive and often becomes tedious for the quick evaluation of binding affinities.

**Figure 1:**
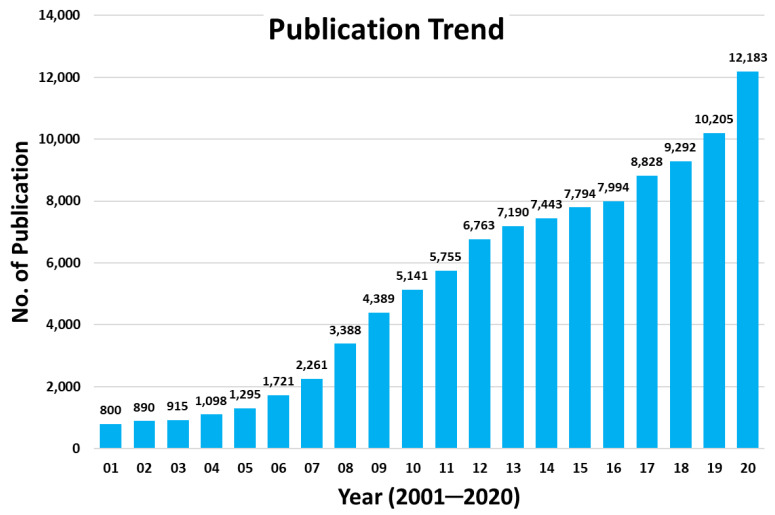
Trend of publications from docking studies in the last two decades obtained from PubMed (https://www.ncbi.nlm.nih.gov/pmc/, accessed on 25 January 2021).

Currently, the field of computer-aided drug-design (CADD) is dominated by molecular docking approach, for which scoring functions are used to identify and rank possible binding poses of a ligand in a binding pocket. As per the records in Swiss Institute of Bioinformatics, there are 57 tools and 20 web services available for molecular docking (Click2Drug: https://www.click2drug.org, accessed on 28 April 2021). The classic scoring functions are broadly divided into three classes—MM force field, empirical scoring and knowledge-based scoring functions. The knowledge-based scoring function incorporates binding modes present in the training dataset but is also accompanied with a requirement of a considerable database having high-quality empirical structures for training [10,11]. On the other hand, machine learning approach deduces the functional form directly from the data. The functional form for the relationship between the structural features and binding affinity of the protein-ligand complex is not predetermined [12]. There are two types of models generally adopted in protein-ligand docking, namely, “Lock and Key” and “Induced-fit”. For docking, two well-known programs, AutoDock [13] and GOLD [14]. have been used widely because of their easy accessibility [15]. Recently, the program Rosetta [16] has also become popular. Figure 1 is plotted based on the data retrieved from PubMed, and depicts the steadily increasing trend of publication on docking studies during the past two decades.

Research in medicinal chemistry is heavily dependent on docking tools and scoring functions, but it has been observed that these scoring functions can result in accuracies anywhere between 0–92.66% and thus their reliability remains a major concern [15]. However, the introduction of experimentally derived crystal structure geometries has proven to improve the success rate to as high as 99% [17]. For induced-fit modelling, the flexibility of protein affects both the scoring and ranking of the best poses. This arises from the additional burden of accurately analysing protein conformational free energy changes apart from ligand binding free energies [18]. Thus, the performances of docking and scoring functions are assessed based on two quantities. First, the reproduction of binding poses of the ligands to that present in the protein complex crystal structures, in which docking is considered accurate only if the heavy atom root mean square displacement (RMSD) is ≤2.0 Å from the localization of crystalized ligand for the top ranked poses [19]. Second, the enrichment factor (EF), which validates docking and scoring algorithms by examining them after screening [19]. For a given percentile limit, higher the EF value better the scoring function. EF studies require large dataset like A Database of Useful Decoys: Enhanced (DUD-E) [20], Maximum Unbiased Validation (MUV) [21] and Comparative Assessment of Scoring Function (CASF) [22], which contain actives and decoys for a diverse set of proteins. In general, the docking and scoring functions are assessed using these two parameters and seldom based on the binding affinity. Additionally, the employment of deep-learning approach [23,24], the graph matching method [25], followed by the traditional docking process have shown to improve the accuracy of protein-ligand binding mode. However, this approach could be target specific [26]. A comparative study by Wang et al. evaluated 11 scoring functions with 100 protein-ligand complexes by accessing their ability to reproduce the binding conformations and affinities [27]. Out of those 11 scoring functions only four resulted a ranking correlation of 50–70% for predicting the binding affinities for the complexes with RMSD criterion of ≤2 Å [27]. Another recent study using a large set of scoring functions suggests that the choice of scoring functions highly depends on the environment of the active site of a target [28]. The use of CASF demonstrated that the current docking tools have promising “docking power” in comparison to “scoring power”, “ranking power” and “screening power” [22].

Recently, the quantum crystallographic (QCr) approach—kernel energy method (KEM) [29]—has been successfully employed for estimating the protein-ligand interaction energies in a simplified, accurate and yet faster way in comparison to the other similar fragment-based approaches [30]. Calculating the ab-initio density matrices using any chemical model present within the quantum chemistry and using crystallographic coordinates is the forte of KEM [31]. In proteins or their complexes, the fragments (a.k.a. kernels) can be as small as one amino acid or a ligand molecule. Thus, the desired energy is estimated at a considerably reduced computational cost. Since its inception, KEM has been applied to a large variety of systems like peptides [29], DNA [32], RNA [33], and proteins [34,35]. Huang et al. have also calculated the interaction energies of aminoglycoside drugs with the target ribosomal A site of RNA as well as the hydrogen bonding interactions within the double stranded RNA [36]. Based on their numerous studies, Massa et al. have demonstrated that the accuracy of energies obtained using KEM is independent of the basis functions and the MP2 method [37,38] provides the best results in comparison to HF and DFT.

Alongside KEM, counterpoise (CP)-corrected energy calculation [39], which accounts for the basis set superposition errors (BSSE) [40], is essential for the accurate estimation of interaction energy (IE) of a hydrogen-bonded complex [41,42]. IE thus calculated provides both CP-corrected and raw (uncorrected) energy values and their average value provide a good estimation of energy for a hydrogen-bonded complex system [42]. In our recent study on exploration of potent ligands for proteins based on the IEs for protein-ligand complexes containing both polar and non-polar interactions, the use of CP corrected KEM (hereafter referred to as KEM-CP) provided accurate results [43].

The aforementioned studies clearly indicate that the requirement of a lead structure is imperative for drug discovery and even for molecular docking when predicting the potential of a ligand towards the formation of its complex with a specific target. Therefore, in this study, we employ the KEM-CP approach [43] for predicting the lead structures based on their IE. For this, we consider three protein complexes with resolutions ranging from ultra-high to standard to low and binding pocket environment ranging from highly hydrophilic to moderately hydrophobic to highly hydrophobic in nature (Table 1). The protein complex structures harvested from RCSB PDB, are (a) 2-(4-bromo-2-fluorobenzylthiocarbamoyl)-5-fluorophenoxyacetic acid (IDD594) bound human aldose reductase (hAR-IDD594), (b) O6-cyclohexylmethoxy-2-(4′-sulphamoylanilino) purine (NU6102) bound cyclin dependent kinase 2 (CDK2-NU6102, PDB ID-1H1S) and (c) 1-chloro-6-(4-hydroxyphenyl)-2-napthol (4NA) bound estrogen receptor β (ERβ -4NA, PDB ID-1YY4). Subsequently, we compare the scoring functions GoldScore [14], ChemScore [44,45] and ChemPLP [46], as implemented in GOLD [14] and utilize the superior scoring function GoldScore for docking some of the known ligands (Schemes S1–S3, Supplementary Materials) with structures similar to their ligands present in the respective complex structures. Thereby, for all the ligands, irrespective of their fitting scores, we select various types of poses predicted via docking and estimate their IEs using KEM-CP. Finally, based on their fitting scores (from GoldScore) and binding IEs (from KEM-CP) we predict the lead structure(s). We also take into account the heavy atom RMSD of the poses with respect to the crystal structure of the ligand present in the complex for predicting the accurate lead structures. Moreover, we compare the ranking of the ligands based on both the fitness scores and the IEs with the ranking based on the reported experimental IC_50_ values, except for hAR-IDD594 complex. Thus, we demonstrate the versatility of KEM-CP and its accuracy over the well-known scoring tool GoldScore.

## 2. Results

### 2.1. Case of hAR-IDD594

In this case, for the five ligands, GoldScore predicts two major types of orientation of poses (namely Type 1 and Type 2) as the best poses (Figure S1 and Table S1, Supplementary Materials). For all the five ligands for both types of poses the IEs are calculated for the best poses (highest fitting score, Table 2) and the RMSD of the poses are compared with the crystal geometry of IDD594 (Table 3). Although the Type 2 poses of the ligands 24 and 25 secure rank 1, their IE values are unfavourable and the corresponding RMSDs are significantly large (>2 Å). Similar trend is noticed for the ligands 10, 16 and 19 with Type 2 pose and with lower rank. Interestingly, the Type 1 pose of the five ligands results in the least RMSD with the crystal geometry of IDD594 and their IE values compare very well with that of the IDD594 crystal geometry (−104.79 kCal·mol^−1^). The pairwise IEs for all the docked poses are listed in Tables S2–S6, Supplementary Materials.

**Figure 2:**
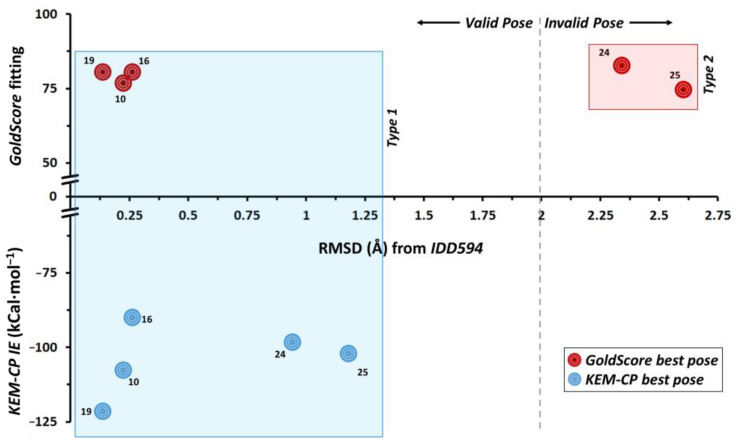
Distribution of RMSD (Å) vs. fitting score from GoldScore and vs. KEM-CP based average IE (Table 2 and Table 3) of hAR ligands.

The results depicted in Figure 2 suggest that KEM-CP approach predicts the best poses for all the five ligands of hAR with RMSDs of <1.2 Å. Whereas GoldScore could predict correct poses only for the ligands 10, 16 and 19 with RMSDs of <0.3 Å.

### 2.2. Case Study of CDK2-NU6102

**Figure 3:**
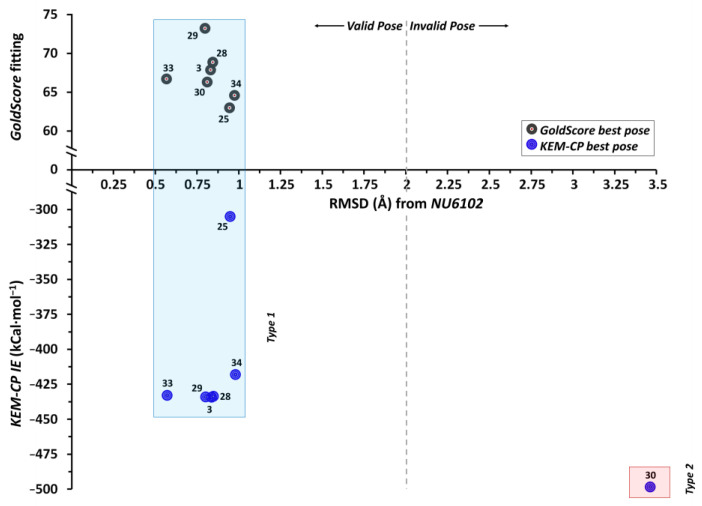
Distribution of RMSD (Å) vs. fitting score from GoldScore and vs. KEM-CP based average IE of CDK2 ligands as listed in Table 4 and Table 5.

In this case, the majority of the 30 poses generated for each of the seven ligands using GoldScore belong to three types of poses as shown in Figure S2, Supplementary Materials. The populations of each type and the overall populations of all three types (≥70%, except for ligand 33 with poor IC_50_ value) for each ligand are listed in Table S7, Supplementary Materials. The average IEs along with the IC_50_ values [48], fitting scores and the corresponding ranks are listed in Table 4. While for ligand 28, none of the poses belongs to Type 2, for ligand 29, only one pose belongs to Type 2. The IE calculation for the Type 3 pose of ligand 34 was failed even using lower basis sets, possibly due to the unfavourable geometry of this particular pose. For all the seven ligands, GoldScore predicts Type 1 pose as the best pose. However, to avoid conformational biasness, we calculate the KEM-CP based IEs for all three types of poses. Interestingly, KEM-CP also predicts Type 1 as the best pose, except for ligand 30, for which Type 2 is predicted as the best pose. Further, the comparison of RMSDs of these three types of poses of the ligands with that of the crystal geometry suggests that for all the seven ligands the Type 1 is the most favourable pose, which has least RMSD of <1 Å (Table 5, Figure 3). Moreover, among the three types of poses, the ligands with Type 1 pose have average IEs closest to that of the crystal geometry of NU6102 ligand (−444.7 kCal·mol^−1^, Table S8, Supplementary Materials). Whereas, both Type 2 and Type 3 poses have RMSDs of >2.7 Å and >3.3 Å, respectively and the IEs of most of these ligands differ by a large to the IE of the NU6102 ligand. The pairwise IEs for all the docked poses including the crystal geometry are listed in Tables S8–S14, Supplementary Materials.

**Figure 4:**
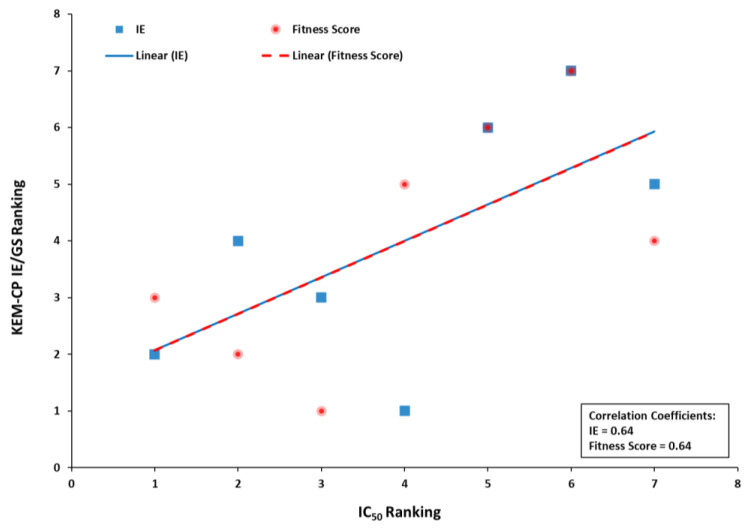
Plot showing IC_50_ ranking distribution with the KEM-CP IE and GoldScore fitness score rankings for the best predicted poses as listed in Table S15, Supplementary Materials.

Further, given a similar prediction by both KEM-CP and GoldScore for a good number of ligands, in this case, we rank the ligands based on the result from these two approaches and compared to the ranking based on the experimental IC_50_ values (Table S15, Supplementary Materials). Interestingly, both the KEM-CP- and the GoldScore-based rankings for the best poses correlate very well (64%) with the rankings based on the experimental IC_50_ values (Table S15, Supplementary Materials and Figure 4). While focusing only on the pose Type 1 (Table S16 and Figure S3, Supplementary Materials), which is grossly predicted as the best pose by KEM-CP and GoldScore, the correlation between the IE and the IC_50_ value has further improved to 71%. Such an excellent correlation of the predicted ranking to the experimental ranking of the ligands of CDK2 suggests that both the approaches, KEM-CP and the GoldScore, perform similarly for proteins with hydrophilic active site.

### 2.3. Case of ERβ-4NA

**Figure 5:**
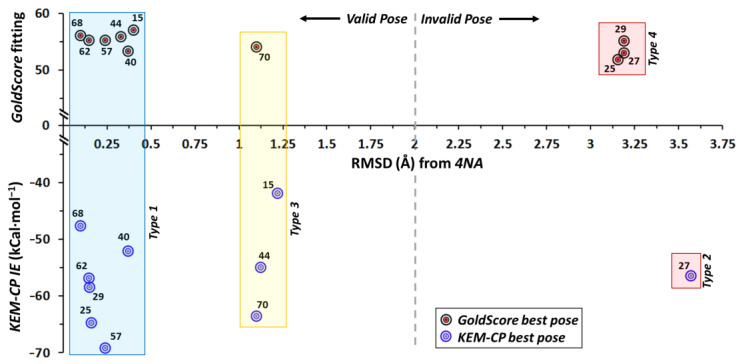
Distribution of RMSD (Å) vs. fitting score from GoldScore and vs. KEM-CP based average IE of ERβ ligands as listed in Table 6 and Table 7.

In this case, the 30 poses generated for each of the 10 ligands using GoldScore are distributed into four types of poses (Figure S4, Supplementary Materials). The distribution of populations of the four types of poses for each ligand are listed in Table S17 (Supplementary Materials). The average IEs along with the IC_50_ values [49], fitting score and the corresponding ranks are listed in Table 6. For ERβ, the GoldScore predicts Type 1 pose as the best pose for majority of the ligands (six: 15, 40, 44, 57, 62 and 68), Type 4 as the best pose for three ligands (25, 27 and 29), Type 3 as the best pose for ligand 70 only and none from Type 2. Interestingly, KEM-CP also predicts Type 1 pose as the best pose for six ligands (25, 29, 40, 57, 62, and 68), Type 3 as the best pose for three ligands (15, 44, 70), Type 2 as the best pose for ligand 27 only and none from Type 4. Further, the comparison of RMSDs (Table 7) of these four types of poses of the ligands with the crystal geometry of 4NA suggests that Type 1 (RMSD < 0.4 Å) and Type 3 (RMSD < 1.3 Å) are the correctly predicted poses for these ligands. Moreover, among the four types of poses, the ligands with Type 1 and Type 3 poses have average IEs closest to that of the crystal geometry of 4NA ligand (−53.5 kCal·mol^−1^, Table S18, Supplementary Materials). Both Type 2 and Type 4 poses are found to be the incorrect predictions as their RMSDs are >3 Å and the IEs for most of these ligands differ by a large to the IE of the 4NA. Accordingly, GoldScore predicts the correct pose only for seven ligands (six of Type 1 and one of Type 3) whereas KEM-CP predicts the correct pose for as many as nine out of the ten ligands (six of Type 1 and three of Type 3) as shown in Figure 5. However, both GoldScore and KEM-CP predict Type 1 as the best pose for the ligands 40, 57, 62 and 68 and Type 3 as the best pose for the ligand 70. The pairwise IEs for all the docked poses are provided in Tables S18–S27, Supplementary Materials. It is noteworthy that although both Type 1 and Type 3 poses are considered favourable, with respect to the Type 1 pose the two fused rings of the ligands in Type 3 pose is rotated by 180° along the single bond present between the two aromatic groups.

**Figure 6:**
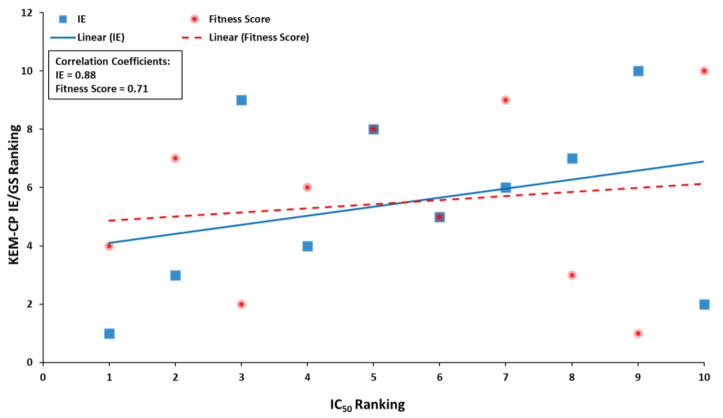
Plot showing IC_50_ ranking distribution with the KEM-CP IE and GoldScore Fitness score rankings for the best predicted poses (data from Table S28).

Further, in this case also, the best pose ligands are ranked based on the average IEs and the fitting scores are compared with the ranking based on their experimental IC_50_ values. For these ten ligands, the correlations of the rankings based on the average IE and the GoldScore to that of the IC_50_ values resulted only 31% and 14%, respectively (Table S28 and Figure S5, Supplementary Materials). However, the ranking based on the average IE and IC_50_ is correlated to the least for the ligands 25 and 68, excluding which the overall correlation for eight ligands has improved to as high as 88%. Likewise, for GoldScore, excluding the ligands 15 and 44 the correlation with IC_50_ has improved to 71% (Table S28, Supplementary Materials and Figure 6). While focusing only on Type 1 pose, the corresponding correlations are 55% and 67%, respectively (Table S29 and Figure S6, Supplementary Materials), whereas, for pose Type 3, the correlations are 48% and 41%, respectively (Table S30 and Figure S7, Supplementary Materials).

## 3. Discussion

Initially, by docking the ligand IDD594 to the ultra-high resolution hAR crystal structure and without supplying any lead structure both qualitatively and quantitatively we confirm that the scoring function GoldScore is indeed superior to the ChemScore and ChemPLP.

In the case of hAR-IDD594, KEM-CP approach could predict the correct pose of the ligands solely based on their IEs as the differences between the IEs of the two types of poses are significantly high (Table 3). Additionally, the corresponding RMSDs correlated extremely well with their IEs, poses with least IE resulted minimum RMSD. However, in the cases of CDK2-NU6102 and ERβ-4NA, the KEM-CP based IEs of the various poses resulted similar values. Therefore, in these two cases, for predicting the correct types of poses, the RMSDs of the best poses predicted by the KEM-CP approach and the GoldScore are compared with the crystal geometries of the corresponding ligands.

Overall, GoldScore predicted correct poses for only three out of five (60%) ligands of hAR, which has moderately hydrophobic active site and seven out of ten (70%) ligands for ERβ with highly hydrophobic active site (Table 8). As expected, for CDK2 with a hydrophilic active site, GoldScore predicts correct poses for all the seven ligands (100%). Interestingly, KEM-CP approach did not show any such environment specificity and it could predict the correct poses for all the five (100%) ligands of hAR, for nine out of the ten (90%) ligands of ERβ and for six out of the seven (86%) ligands of CDK2 (Table 8).

For both ERβ and CDK2, the ranking comparison emphasizes that the prediction of the lead structures by the KEM-CP approach is as good as the experimental IC_50_ values, whereas the GoldScore approach could predict well only for the CDK2. Such an excellent correlation of the predicted ranking to the experimental ranking of the ligands of CDK2 suggests that both the approaches, KEM-CP and the GoldScore, perform similarly for proteins with hydrophilic active site. For hAR, such a comparison did not result a meaningful correlation, possibly due to the small number of ligands and inadequate experimental measurements (without error analysis).

Overall, a better correlation of ranking is observed for the CDK2 in comparison to the ERβ. This is because the resolution of the crystal structure of CDK2 is better (2.0 Å) than that of ERβ (2.7 Å). Moreover, CDK2 has hydrophilic environment, for which GoldScore performs better.

## 4. Materials and Methods

### 4.1. Selection of Complex Structures and Preparation of the Targets and Their Ligands

The rationale behind the selection of the three protein complexes with distinct active site environments is summarized below:

(1) *hAR*-*IDD594*: The ultra-high resolution (0.66 Å) complex structure was previously considered by some of us for studying protein-ligand interactions and for benchmarking KEM-CP approach against *MOE* scoring function [43]. Here, once again, we consider this complex structure with moderately hydrophobic active site environment for benchmarking KEM-CP approach against GoldScore.
(2) *CDK2*-*NU6102*: This standard resolution (2.0 Å) complex structure has a hydrophilic environment in its active site and previous report [28] suggests that GoldScore provides better results for such systems. Therefore, we select this system to check the superiority of KEM-CP over GoldScore.
(3) *ERβ*-*4NA*: This complex structure consists of a hydrophobic (or lyophilic) active site and reported at a low resolution of 2.7 Å. As reported earlier, GoldScore fails to rank potent ligand accurately for proteins with hydrophobic environments [28] (Table 1) and hence IE study on such a system provides us an opportunity to test the potentiality of KEM-CP approach.

Such selections allowed us to investigate distinct protein active site environments ranging from highly hydrophilic (CDK2) to moderately hydrophobic (hAR) to highly hydrophobic (ERβ) in nature. Moreover, these target systems provide an opportunity to investigate structures of varying resolution ranging between 0.66 Å to 2.7 Å (Table 1). 

The retrieved protein structures from RCSB PDB were prepared for docking by selecting the major conformer (wherever multiple conformers were present) using *pdbset* module of *CCP4* [50], which was also used to remove the solvents including water molecules. The atomic coordinates were then fed into GOLD. Subsequently, the metal ions (none was at the active site) were removed and the H-atoms, protonation state and tautomerization were assigned. The hydrophobicity of the protein active site was calculated using *SiteMap* (SiteMap, Schrödinger, LLC, New York, NY, USA, 2020) [47] and the corresponding values are listed in Table 1.

The chemical diagrams of the ligands [7,48,49] (Schemes S1–S3, Supplementary Materials) were drawn using ChemDraw (PerkinElmer Informatics, Waltham, MA, USA, 2018) and their structures were further optimized using Gaussian09 (Gaussian, Inc., Wallingford, CT, USA, 2009) at the *MP2*/*6*-*311G*(*d*,*p*) level of theory. The rationales for the ligand selections are discussed below.

- For hAR, we select five ligands (including IDD594) with similar scaffolds (Scheme S1, Supplementary Materials) as reported by Ferrari et al. [7].
- For CDK2, seven ligands (Scheme S2, Supplementary Materials) with best experimental IC_50_ values are chosen from the study by Hardcastle et al. [48]. Despite having lower IC_50_ value the ligand 33 is retained in the list because it is an isomer of NU-6102 (with sulphonamide substitution on phenyl ring at the *meta* position instead of the *ortho* position). This provides an additional opportunity to explore the applicability of KEM-CP approach for the isomers.
- For ERβ, although Mewshaw et al. [49] have studied ~70 ligands with IC_50_ values ranging from 2.0 μM–0.5 nM, an IC_50_ cut-off of 3.0 nM resulted in 24 ligands, out of which we select 10 ligands to include various kinds of functional groups (Scheme S3, Supplementary Materials) in our analysis.

### 4.2. Scoring Function and Docking Studies

The accuracy of each of the scoring functions, GoldScore [14], ChemScore [44,45] and ChemPLP [46], is tested by docking the ligand IDD594 to the active site of the ultra-high resolution hAR-IDD594 complex structure (see Section S1.1, Supplementary Materials). Thereby, the poses with rank 1 of the IDD594 ligand as provided by the three scoring functions are compared among themselves (Figure S8, Supplementary Materials) as well as with the crystal geometry of IDD594 (Figure S9, Supplementary Materials). The qualitative comparison suggests that the pose predicted by GoldScore is closest to its crystal geometry. Further, to bring the quantitative comparison, in each case, we calculate the IE using KEM-CP approach as discussed in the following section. The corresponding pairwise IEs are listed in Tables S31–S34, Supplementary Materials. Thus, the total IEs of hAR with the best poses of IDD594 as predicted by the three scoring functions and with the crystal geometry of IDD594 along with the RMSDs of the poses with respect to the crystal geometry are compared (Table S35, Supplementary Materials). The comparisons suggest that the pose predicted by the GoldScore has the least RMSD and minimum IE (Table S35, Supplementary Materials). Therefore, we conclude that GoldScore is superior among these three scoring functions, which was also pointed out by Xu et al. [28]. Subsequently, the ligands (Schemes S1–S3, Supplementary Materials) selected in this study were docked into the target protein structures using GoldScore. The binding pocket was defined by selecting a sphere of radius 5 Å with one of the residues located deep inside the active site as the centre. The central residues (Trp111 for hAR, Leu354 for ERβ and Phe80 for CDK2) are highlighted in blue in Figure S10, Supplementary Materials. The docking was performed based on the “Lock and Key” model without supplying any lead structure as input and the ligands were allowed to be highly flexible. Thereby, 30 poses were generated for each ligand. The fitness scores thus obtained from the docking are unit less and higher the fitness score better the binding affinity of the ligand to the target. The 30 poses are then grouped into certain types of orientations with a significant number of poses within a given orientation type. Subsequently, using KEM-CP, the IEs are calculated for the best ranked posed from each type of orientations. 

### 4.3. KEM-CP Interaction Energy Calculation and Kernel Selection

The first step of KEM is to fragment the macromolecule into small pieces call kernels such that every atom of the macromolecule belongs to one and only one kernel (Figure S11, Supplementary Materials) [29]. Subsequently, the kernels are capped at the point of fragmentation using H-atoms to preserve the valency. The quantum calculations are performed on the single kernels and double kernels (pairs) and their contributions are then summed up to get the total molecular energy as follows (Equation (1)):

(1) $$E_{total}=\overset{n-1}{\underset{m=1}{\sum }}\left(\overset{n-m}{\underset{\begin{array}{c} i=1\\ j=i+m \end{array}}{\sum }}E_{ij}\right)-\left(n-2\right)\overset{n}{\underset{i=1}{\sum }}E_{i}$$

where *n* is the number of single kernels, *m*, *i*, *j* are the running numbers, *E_ij_* is the energy of a double kernel and *E_i_* is the energy of a single kernel.

The IE between any two kernels, *I_ij_* is defined as the difference of energy between their double kernel (*E_ij_*) and the constituent single kernels (*E_i_* and *E_j_*), represented as (Equation (2)):

(2) $$I_{ij}=E_{ij}-E_{i}-E_{j}$$

In general, for protein-ligand IE calculation, the ligand is chosen as the first kernel, and each of the residues/solvent present as the immediate neighbour of the ligand are selected as the second kernel. Thus, forming the single and double kernels (pair of kernels), the protein-ligand interaction energy, *I_protein−ligand_* is estimated by the summation of all such kernel pairs as follows (Equation (3)):

(3) $$I_{protein-ligand}=\underset{i\neq j}{\sum }I_{ij}$$

The calculations on the double kernels (dimers) are performed using ‘counterpoise’ keyword in Gaussian09 [51]. after assigning the fragment number to each monomer along with their charge and multiplicity information. 

The KEM-CP calculations are performed on multiple types of orientation of poses of every ligand of hAR (2 types per ligand), CDK2 (3 types per ligand) and ERβ (4 types per ligand) using *MP2/6-311G*(*d*,*p*) level of theory. All the residues identified at the active site, irrespective of their contact with the ligand, are considered for the IE calculations. Thereby, the variation in the protein-ligand interactions due to the conformational changes of the ligands at the active site are monitored. In any case, in the absence of a contact, the KEM-CP correctly predicts a negligible IE.

## 5. Conclusions

Here, we employ the quantum crystallographic approach, KEM-CP, for predicting accurate lead ligand structures for proteins hAR, CDK2 and ERβ. Our study demonstrates an important application of KEM-CP for drug discovery. We select protein systems with active site environments ranging from highly hydrophilic to moderate to highly hydrophobic and with structural resolutions ranging from ultra-high (0.66 Å, hAR-IDD594) to standard (2 Å, CDK2-NU6102) to low (2.7 Å, ERβ-4NA) for exploring the versatility of KEM-CP approach. Our comparative study based on the hAR-IDD594 complex structure confirms that GoldScore is indeed superior to the other two scoring functions as implemented in the package GOLD. However, the results based on the KEM-CP approach in conjunction with the molecular docking demonstrate that not necessarily the top ranked pose predicted by the GoldScore is the best pose for a given target. Comparison of the fitting score and the IE based results with the RMSDs of the ligands w.r.t. their crystal geometries allowed identifying the accurate lead structures. Further, the ranking of the ligands based on our results correlated well with that of the experimental IC_50_ values. Although, the GoldScore is active site environment specific, KEM-CP approach shows neither such environment specificity nor any dependency on the structural resolutions. Moreover, besides its efficiency and quickness, KEM-CP calculations are simple and can be performed economically. KEM-CP can also be used for exploring the potent ligands of a system for which only an apo crystal structure or even a simulated structure is known. These clearly demonstrate the versatility and simplicity of the KEM-CP approach. Nevertheless, further such studies on some more systems would be necessary to judge the enhanced capability of KEM-CP. Moreover, the usefulness of KEM-CP based accurate poses, especially their orientations, for the EF based benchmarking using DUD-E, MUV, CASF etc. could be worth exploring in the future. Lastly, it is evident from our analysis that the application of KEM-CP approach along with docking studies may ease the process of virtual screening of potent ligands—the bottleneck of drug discovery research.

## Figures and Tables

**Table 1:** Details of the target protein structures.

Protein Complexes	hAR-IDD594	CDK2-NU6102	ERβ-4NA
PDB ID	1US0	1H1S	1YY4
Resolution	0.66 Å	2.0 Å	2.7 Å
*SiteMap* Score [47] *			
Hydrophobic	3.0	1.4	4.4
Hydrophilic	0.7	1.0	0.3
Balance **	4.2	1.4	13.3

* Radius of 5.0 Å was used for binding site. ** Balance >6.0 indicates high hydrophobicity and/or likely lipophilicity.

**Table 2:** List of ligands of hAR with their average IEs and GoldScore fitness scores for the two types of poses. The most negative IEs and the best fitness scores from GoldScore are highlighted using (values in bold). The reported experimental IC_50_ [7] values are also listed.

Ligand #	Experimental IC_50_ (nM)	Pose Type 1			Pose Type 2		
		Avg. IE (kCal·mol^−1^)	GoldScore		Avg. IE (kCal·mol^−1^)	GoldScore	
			Fitness Score	Rank		Fitness Score	Rank
10	176	**−107.67**	**76.93**	1	2.35	58.51	19
16	44	**−89.93**	**80.56**	1	−18.68	72.97	4
19	30	**−121.11**	**87.08**	1	18.24	76.64	2
24	7	**−98.39**	77.90	2	−33.42	**82.73**	1
25	6	**−100.45**	73.76	2	−19.97	**74.75**	1

**Table 3:** Comparison of RMSDs of both types of poses with respect to the crystal geometry of IDD594.

Ligand #	Pose Type 1		Pose Type 2	
	Avg. IE (kCal·mol^−1^)	RMSD Crystal Geometry (Å)	Avg. IE (kCal·mol^−1^)	RMSD Crystal Geometry (Å)
Predicted pose superimposed on crystal geometry (grey)	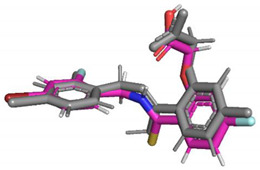		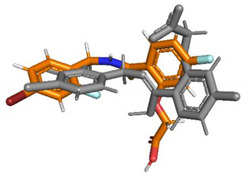	
10	−107.67	0.223	2.35	2.042
16	−89.93	0.263	−18.68	2.563
19	−121.11	0.137	18.24	2.128
24	−98.39	0.943	−33.42	2.342
25	−100.45	1.180	−19.97	2.603

**Table 4:** List of ligands of CDK2 with their IC_50_ values [48] and average IEs and docking score details for the three types of poses. The most negative IEs and the best fitness scores are highlighted (values in bold).

Ligand #	Experimental IC_50_ (nM)	Pose Type 1			Pose Type 2			Pose Type 3		
		Avg. IE (kCal·mol^−1^)	GoldScore		Avg. IE (kCal·mol^−1^)	GoldScore		Avg. IE (kCal·mol^−1^)	GoldScore	
			Fitness Score	Rank		Fitness Score	Rank		Fitness Score	Rank
3	5.4 ± 1.0	**−434.26**	**67.87**	1	−422.08	63.07	2	−299.76	60.32	3
25	69 ± 1	**−304.97**	**62.98**	1	−258.26	59.74	2	−219.28	55.47	3
28 *	7.0 ± 0.1	**−433.67**	**68.88**	1	-	-	-	−334.16	56.93	10
29	56 ± 20	**−434.11**	**73.24**	1	−376.89	59.73	4	−376.57	55.92	11
30	63 ± 7	−398.53	**66.33**	1	**−498.35**	66.16	3	−362.61	54.99	12
33	210 ± 40	**−432.88**	**66.71**	1	−414.07	63.00	4	−317.21	56.66	14
34	64 ± 33	**−418.00**	**64.60**	1	−334.12	59.39	2	Failed	55.37	6

* None of the poses belonged to Type 2.

**Table 5:** Comparison of RMSDs of the three types of predicted poses with respect to the crystal geometry of NU6102.

Ligand #	Pose Type 1		Pose Type 2		Pose Type 3	
	Avg. IE (kCal·mol^−1^)	RMSD with Crystal Geometry (Å)	Avg. IE (kCal·mol^−1^)	RMSD with Crystal Geometry (Å)	Avg. IE (kCal·mol^−1^)	RMSD with Crystal Geometry (Å)
Predicted pose superimposed on crystal geometry (grey)	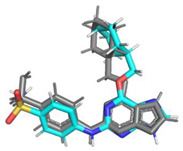		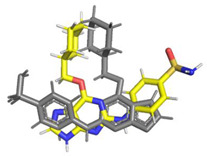		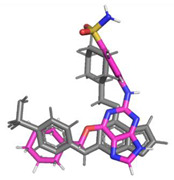	
3	−434.26	0.832	−422.08	4.072	−299.76	3.212
25	−304.97	0.944	−258.26	4.379	−219.28	2.947
28	−433.67	0.844	-	-	−334.16	3.221
29	−434.11	0.798	−376.89	3.797	−376.57	2.752
30	−398.53	0.811	−498.35	3.454	−362.61	2.950
33	−432.88	0.567	−414.07	3.680	−317.21	2.908
34	−418.00	0.975	−334.12	3.330	Failed	2.875

**Table 6:** List of ligands of ERβ with their IC_50_ values [49] and average IEs and docking score details for the four types of poses. The most negative IEs and the best fitness scores are highlighted (values in bold).

Ligand #	Experimental IC_50_ (nM)	Pose Type 1			Pose Type 2			Pose Type 3			Pose Type 4		
		Avg. IE (kCal·mol^−1^)	GoldScore		Avg. IE (kCal·mol^−1^)	GoldScore		Avg. IE (kCal·mol^−1^)	GoldScore		Avg. IE (kCal·mol^−1^)	GoldScore	
			Fitness Score	Rank		Fitness Score	Rank		Fitness Score	Rank		Fitness Score	Rank
15	2.52 ± 1.3	−40.33	**57.10**	1	−30.44	52.58	3	**−41.90**	55.11	2	−25.86	51.85	4
25	2.8 ± 0.1	**−64.69**	50.76	3	−54.59	49.80	6	−57.96	50.73	4	−48.19	**51.87**	1
27	2.3 ± 0.1	−49.90	49.68	7	**−56.42**	50.17	3	−43.79	50.02	5	−50.99	**53.05**	1
29	1.4 ± 0.6	**−58.45**	52.28	3	−37.37	51.88	5	−42.65	51.99	4	−50.74	**55.15**	1
40	1.6 ± 0.7	**−52.07**	**53.38**	1	−52.03	49.54	7	−49.17	52.11	3	−41.80	52.68	2
44	2.3 ± 1.7	−53.64	**55.86**	1	−42.58	49.53	5	**−54.89**	54.53	3	−24.37	52.34	4
57	0.5 ± 0.5	**−69.15**	**55.29**	1	−36.44	47.02	9	−51.64	52.72	3	−21.68	49.41	5
62	2.1 ± 0.9	**−56.79**	**55.28**	1	−26.68	53.51	5	−40.83	55.12	3	−38.80	49.52	11
68	1.2 ± 0.7	**−47.56**	**56.13**	1	−2.31	47.19	9	−39.92	55.18	3	−19.58	45.38	11
70	1.1 ± 1.6	−47.88	53.93	2	−38.95	51.50	7	**−63.55**	**54.13**	1	−28.36	50.32	10

**Table 7:** Comparison of RMSDs of the four types of predicted poses with respect to the crystal geometry of 4NA.

Ligand #	Pose Type 1		Pose Type 2		Pose Type 3		Pose Type 4	
	Avg. IE (kCal·mol^−1^)	RMSD (Å)	Avg. IE (kCal·mol^−1^)	RMSD (Å)	Avg. IE (kCal·mol^−1^)	RMSD (Å)	Avg. IE (kCal·mol^−1^)	RMSD (Å)
Predicted pose superimposed on crystal geometry (grey)	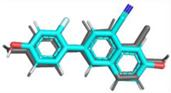		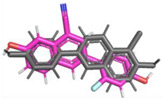		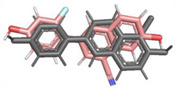		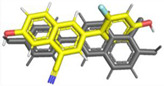	
15	−40.33	0.399	−30.44	3.526	−41.90	1.219	−25.86	3.185
25	−64.69	0.161	−54.59	3.430	−57.96	1.100	−48.19	3.155
27	−49.90	0.276	−56.42	3.570	−43.79	1.027	−50.99	3.189
29	−58.45	0.149	−37.37	3.390	−42.65	0.978	−50.74	3.190
40	−52.07	0.371	−52.03	3.452	−49.17	1.044	−41.80	3.193
44	−53.64	0.329	−42.58	3.515	−54.89	1.122	−24.37	3.221
57	−69.15	0.239	−36.44	3.499	−51.64	1.117	−21.68	3.227
62	−56.79	0.147	−26.68	3.420	−40.83	1.040	−38.80	3.380
68	−47.56	0.099	−2.31	3.393	−39.92	0.992	−19.58	3.255
70	−47.88	0.153	−38.95	3.147	−63.55	1.100	−28.36	3.434

**Table 8:** Summary of lead structure prediction by GoldScore and KEM-CP.

Protein	Active Site Environment	No. of Ligands	No. of Ligands Predicted Correctly By		% of Ligands Predicted Correctly By	
			GoldScore	KEM-CP	GoldScore	KEM-CP
**hAR**	Moderately hydrophobic	5	3	5	60	100
**CDK2**	Highly hydrophilic	7	7	6	100	86
**ERβ**	Highly hydrophobic	10	7	9	70	90

## Supplementary Materials

The following information are available online at. Scheme S1: Chemical structures of the five ligands docked in hAR. Scheme S2: Chemical structures of the seven ligands docked in CDK2. Scheme S3: Chemical structures of the ten ligands docked in ERβ. Figure S1: Two major types of orientations of the IDD594 ligand at the binding site. Figure S2: Three major types of orientations of the ligand NU6102 of CDK2 at the binding site and their overlay diagrams. Figure S3: Plot showing IC_50_ ranking distribution with the KEM-CP IE and GoldScore Fitness score rankings for pose Type 1 (from Table S16). Figure S4: Four major types of orientations of the ligand 4NA of ERβ at the binding site and their overlay diagrams. Figure S5: Plot showing correlations of rankings based on the average KEM-CP IE and GoldScore fitness score with the ranking based on IC_50_ values for best poses of all 10 ligands (data from Table S28). Figure S6: Plot showing correlations of rankings based on the average KEM-CP IE and GoldScore fitness score with the ranking based on IC_50_ values for pose Type 1 (data from Table S34). Figure S7: Plot showing correlations of rankings based on the average KEM-CP IE and GoldScore fitness score with the ranking based on IC_50_ values for pose Type 3 (data from Table S35). Figure S8: Best poses of IDD594 in hAR using different scoring functions. Figure S9: Comparison of IDD594 poses generated by different scoring functions with respect to the crystal geometry (blue). Figure S10: Views of the active sites of (a) hAR-IDD594, (b) CDK2-NU6102 and (c) ERβ-4NA highlighting the complexed ligand (cyan) and the residues chosen as the active site centre (blue). Figure S11: Representation of the fragmentation of macromolecule as single and double kernels. Table S1: Population distribution of the ligands of hAR belonging to two major types. Tables S2–S6: Interaction energies (kCal·mol^−1^) of hAR with GoldScore poses of ligand 10, 16, 19, 24 and 25, respectively. Table S7: Population distribution of various poses of the ligands of CDK2 belonging to three major types. Tables S8–S14: Interaction energies (kCal·mol^−1^) of CDK2 with GoldScore poses of ligand 3, 25, 28, 29, 30, 33 and 34, respectively. Table S15: Comparison of ranking based on the experimental IC_50_ values, the average IE from KEM-CP and fitting score from GoldScore of the best poses of the CDK2 ligands. Table S16: Comparison of ranking based on the experimental IC_50_ values, the average IE from KEM-CP and fitting score from GoldScore of the Type 1 poses of the CDK2 ligands. Table S17: Population distribution of the 30 poses of the ligands of ERβ belonging to 4 types. Tables S18–S27: Interaction energies (kCal·mol^−1^) of ERβ with GoldScore poses of ligand 15, 25, 27, 29, 40, 44, 57, 62, 68 and 70, respectively. Table S28: Comparison of ranking based on the experimental IC_50_ values, the average IE from KEM-CP and fitting score of the best poses of the ERβ ligands. Table S29: Comparison of ranking based on the experimental IC_50_ values, the average IE from KEM-CP and fitting score of the pose Type 1 of the ERβ ligands. Table S30: Comparison of ranking based on the experimental IC_50_ values, the average IE from KEM-CP and fitting score of the pose Type 3 of the ERβ ligands. Table S31: Interaction energies (kCal·mol^−1^) of hAR-IDD594 complex. Table S32: Interaction energies (kCal·mol^−1^) of hAR with GoldScore pose of ligand 19. Table S33: Interaction energies (kCal·mol^−1^) of hAR with ChemScore pose of ligand 19. Table S34: Interaction energies (kCal·mol^−1^) of hAR with ChemPLP pose of ligand 19. Table S35: Interaction energy (IE) of hAR with IDD594 poses generated by different scoring function and with the crystal geometry of IDD594 and RMSD of the docked poses with respect to the crystal geometry. Section S1.1: Benchmarking of scoring functions.

## Sample Availability

Samples of the compounds are not available from the authors.

## References

[1] Gohlke H., Klebe G. (2002). Approaches to the Description and Prediction of the Binding Affinity of Small-Molecule Ligands to Macromolecular Receptors. Angew. Chem. Int. Ed..

[2] Gilson M.K., Zhou H.-X. (2007). Calculation of Protein-Ligand Binding Affinities. Annu. Rev. Biophys. Biomol. Struct..

[3] Guimarães C.R.W., Boger A.D.L., Jorgensen W.L. (2005). Elucidation of Fatty Acid Amide Hydrolase Inhibition by Potent α-Ketoheterocycle Derivatives from Monte Carlo Simulations. J. Am. Chem. Soc..

[4] Simonson T., Archontis G., Karplus M. (2002). Free Energy Simulations Come of Age: Protein−Ligand Recognition. Acc. Chem. Res..

[5] Guimarães C.R.W., Cardozo M. (2008). MM-GB/SA Rescoring of Docking Poses in Structure-Based Lead Optimization. J. Chem. Inf. Model..

[6] Kollman P.A., Massova I., Reyes C., Kuhn B., Huo S., Chong L., Lee M., Lee T., Duan Y., Wang W. (2000). Calculating Structures and Free Energies of Complex Molecules: Combining Molecular Mechanics and Continuum Models. Acc. Chem. Res..

[7] Ferrari A.M., Degliesposti G., Sgobba M., Rastelli G. (2007). Validation of an Automated Procedure for the Prediction of Relative Free Energies of Binding on a Set of Aldose Reductase Inhibitors. Bioorganic Med. Chem..

[8] Barreiro G., Guimarães C.R.W., Tubert-Brohman I., Lyons T.M., Tirado-Rives J., Jorgensen W.L. (2007). Search for Non-Nucleoside Inhibitors of HIV-1 Reverse Transcriptase Using Chemical Similarity, Molecular Docking, and MM-GB/SA Scoring. J. Chem. Inf. Model..

[9] Fidelak J., Juraszek J., Branduardi D., Bianciotto M., Gervasio F.L. (2010). Free-Energy-Based Methods for Binding Profile Determination in a Congeneric Series of CDK2 Inhibitors. J. Phys. Chem. B.

[10] Gohlke H., Hendlich M., Klebe G. (2000). Knowledge-Based Scoring Function to Predict Protein-Ligand Interactions. J. Mol. Biol..

[11] Huang S.-Y., Zou X. (2006). An Iterative Knowledge-Based Scoring Function to Predict Protein–Ligand Interactions: II. Validation of the Scoring Function. J. Comput. Chem..

[12] Ballester P.J., Mitchell J.B.O. (2010). A Machine Learning Approach to Predicting Protein–Ligand Binding Affinity with Applications to Molecular Docking. Bioinformatics.

[13] Goodsell D.S., Olson A.J. (1990). Automated Docking of Substrates to Proteins by Simulated Annealing. Proteins.

[14] Jones G., Willett P., Glen R.C., Leach A.R., Taylor R. (1997). Development and Validation of a Genetic Algorithm for Flexible Docking. J. Mol. Biol..

[15] Chen Y.-C. (2015). Beware of Docking!. Trends Pharmacol. Sci..

[16] Leaver-Fay A., Tyka M.D., Davis I.W., Cooper S., Treuille A., Mandell D.J., Richter F., Ban Y.-E.A., Fleishman S.J., Corn J.E. (2011). Rosetta3: An Object-Oriented Software Suite for the Simulation and Design of Macromolecules. Methods Enzymol..

[17] Davis I.W., Baker D. (2009). Rosetta Ligand Docking with Full Ligand and Receptor Flexibility. J. Mol. Biol..

[18] Sotriffer C.A. (2011). Accounting for Induced-Fit Effects in Docking: What is Possible and What is Not?. Curr. Top. Med. Chem..

[19] Huang S.-Y., Grinter S.Z., Zou X. (2010). Scoring Functions and their Evaluation Methods for Protein-Ligand Docking: Recent Advances and Future Directions. Phys. Chem. Chem. Phys..

[20] Mysinger M.M., Carchia M., Irwin J.J., Shoichet B.K. (2012). Directory of Useful Decoys, Enhanced (DUD-E): Better Ligands and Decoys for Better Benchmarking. J. Med. Chem..

[21] Tiikkainen P., Markt P., Wolber G., Kirchmair J., Distinto S., Poso A., Kallioniemi O. (2009). Critical Comparison of Virtual Screening Methods against the MUV Data Set. J. Chem. Inf. Model..

[22] Su M., Yang Q., Du Y., Feng G., Liu Z., Li Y., Wang R. (2019). Comparative Assessment of Scoring Functions: The CASF-2016 Update. J. Chem. Inf. Model..

[23] Morrone J.A., Weber J.K., Huynh T., Luo H., Cornell W.D. (2020). Combining Docking Pose Rank and Structure with Deep Learning Improves Protein–Ligand Binding Mode Prediction over a Baseline Docking Approach. J. Chem. Inf. Model..

[24] Gomes P.D.S.F.C., Da Silva F., Bret G., Rognan D. (2017). Ranking Docking Poses by Graph Matching of Protein-Ligand Interactions: Lessons Learned from the D3R Grand Challenge 2. J. Comput. Aided Mol. Des..

[25] Jiménez-Luna J., Cuzzolin A., Bolcato G., Sturlese M., Moro S. (2020). A Deep-Learning Approach toward Rational Molecular Docking Protocol Selection. Molecules.

[26] Shen C., Hu Y., Wang Z., Zhang X., Pang J., Wang G., Zhong H., Xu L., Cao D., Hou T. (2020). Beware of the Generic Machine Learning-Based Scoring Functions in Structure-Based Virtual Screening. Brief. Bioinform..

[27] Wang R., Lu Y., Wang S. (2003). Comparative Evaluation of 11 Scoring Functions for Molecular Docking. J. Med. Chem..

[28] Xu W., Lucke A.J., Fairlie D.P. (2015). Comparing Sixteen Scoring Functions for Predicting Biological Activities of Ligands for Protein Targets. J. Mol. Graph. Model..

[29] Huang L., Massa L., Karle J. (2005). Kernel Energy Method Illustrated with Peptides. Int. J. Quantum Chem..

[30] Řezáč J., Salahub D.R. (2009). Multilevel Fragment-Based Approach (MFBA): A Novel Hybrid Computational Method for the Study of Large Molecules. J. Chem. Theory Comput..

[31] Massa L., Matta C.F. (2017). Quantum Crystallography: A Perspective. J. Comput. Chem..

[32] Huang L., Massa L., Karle J. (2005). Kernel Energy Method: Application to DNA. Biochemistry.

[33] Huang L., Massa L., Karle J. (2006). The Kernel Energy Method: Application to a tRNA. Proc. Natl. Acad. Sci. USA.

[34] Huang L., Massa L., Karle J. (2005). Kernel Energy Method: Application to Insulin. Proc. Natl. Acad. Sci. USA.

[35] Huang L., Massa L. (2012). Quantum Kernel Applications in Medicinal Chemistry. Future Med. Chem..

[36] Huang L., Massa L., Karle J. (2007). Drug Target Interaction Energies by the Kernel Energy Method in Aminoglycoside Drugs and Ribosomal A Site RNA Targets. Proc. Natl. Acad. Sci. USA.

[37] Frisch M.J., Head-Gordon M., Pople J.A. (1990). A Direct MP2 Gradient Method. Chem. Phys. Lett..

[38] Head-Gordon M., Pople J.A., Frisch M.J. (1988). MP2 Energy Evaluation by Direct Methods. Chem. Phys. Lett..

[39] Boys S.F., Bernardi F. (1970). The Calculation of Small Molecular Interactions by the Differences of Separate Total Energies. Some Procedures with Reduced Errors. Mol. Phys..

[40] Simon S., Duran M., Dannenberg J.J. (1996). How does Basis Set Superposition Error Change the Potential Surfaces for Hydrogen-Bonded Dimers?. J. Chem. Phys..

[41] Halkier A., Klopper W., Helgaker T., Jorgensen P., Taylor P.R. (1999). Basis Set Convergence of the Interaction Energy of Hydrogen-Bonded Complexes. J. Chem. Phys..

[42] Brauer B., Kesharwani M.K., Martin J.M.L. (2014). Some Observations on Counterpoise Corrections for Explicitly Correlated Calculations on Noncovalent Interactions. J. Chem. Theory Comput..

[43] Mandal S.K., Saha P., Munshi P., Sukumar N. (2017). Exploring Potent Ligand for Proteins: Insights from Knowledge-Based Scoring Functions and Molecular Interaction Energies. Struct. Chem..

[44] Eldridge M.D., Murray C.W., Auton T.R., Paolini G.V., Mee R.P. (1997). Empirical Scoring Functions: I. The Development of a Fast Empirical Scoring Function to Estimate the Binding Affinity of Ligands in Receptor Complexes. J. Comput. Aided Mol. Des..

[45] Baxter C.A., Murray C.W., Clark D.E., Westhead D.R., Eldridge M.D. (1998). Flexible Docking using Tabu Search and an Empirical Estimate of Binding Affinity. Proteins Struct. Funct. Bioinform..

[46] Korb O., Stutzle T., Exner T.E. (2009). Empirical Scoring Functions for Advanced Protein−Ligand Docking with PLANTS. J. Chem. Inf. Model..

[47] Halgren T.A. (2009). Identifying and Characterizing Binding Sites and Assessing Druggability. J. Chem. Inf. Model..

[48] Hardcastle I.R., Arris C.E., Jewsbury P., Menyerol J., Mesguiche V., Newell D.R., Noble M.E.M., Pratt D.J., Wang A.L.-Z., Whitfield† H.J. (2004). N2-SubstitutedO6-Cyclohexylmethylguanine Derivatives: Potent Inhibitors of Cyclin-Dependent Kinases 1 and 2. J. Med. Chem..

[49] Mewshaw R.E., Edsall R.J., Yang C., Manas E.S., Xu Z.B., Henderson R.A., Keith J.C., Harris H.A. (2005). ERβ Ligands. 3. Exploiting Two Binding Orientations of the 2-Phenylnaphthalene Scaffold to Achieve ERβ Selectivity. J. Med. Chem..

[50] Winn M.D., Ballard C.C., Cowtan K.D., Dodson E.J., Emsley P., Evans P.R., Keegan R.M., Krissinel E.B., Leslie A.G.W., McCoy A. (2011). Overview of theCCP4 Suite and Current Developments. Acta Crystallogr. Sect. D Biol. Crystallogr..

[51] Frisch M.J., Trucks G.W., Schlegel H.B., Scuseria G.E., Robb M.A., Cheeseman J.R., Scalmani G., Barone V., Mennucci B., Petersson G.A. (2016). Gaussian09.

