# Supplementary material for: Predicting Accurate Lead Structures for Screening Molecular Libraries: A Quantum Crystallographic Approach
Source: Molecules. 2021 Apr 29;26(9):2605. doi: 10.3390/molecules26092605 (PMC8124947; doi:10.3390/molecules26092605)
Supplement: Supplementary file 1 [file molecules-26-02605-s001.zip › molecules-1193841-supplementary.pdf]

Predicting Accurate Lead Structure for Screening Molecular Libraries: A Quantum Crystallographic Approach

*Suman Kumar Mandal and Parthapratim Munshi**

Chemical and Biological Crystallography, Department of Chemistry, School of Natural Sciences, Shiv Nadar University, Dadri, Uttar Pradesh, INDIA 201314

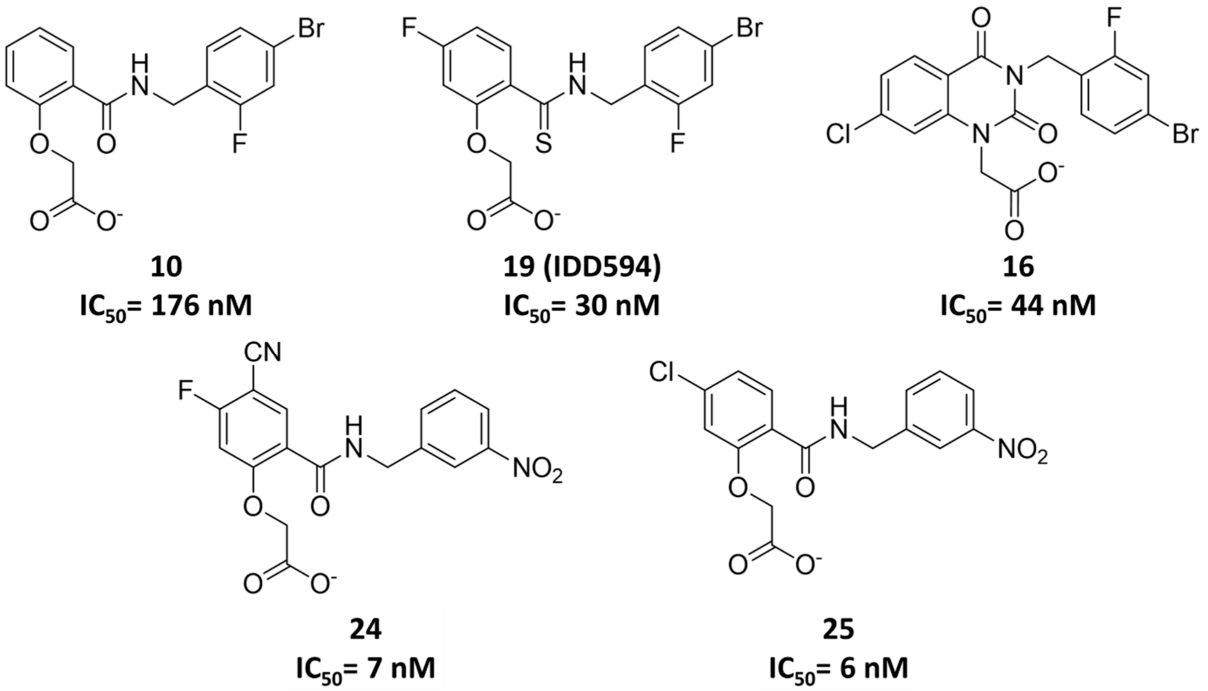

Scheme S1 Chemical structures of the five ligands[1] docked in hAR.

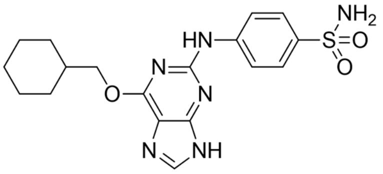

3 (NU6102)
IC_{50}= 5.4 ± 1 nM

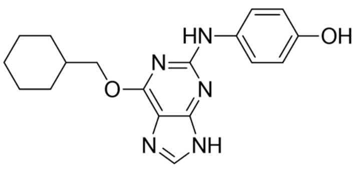

25
IC_{50}= 69 ± 1 nM

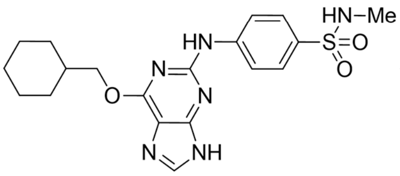

28
IC_{50}= 7.0 ± 0.1 nM

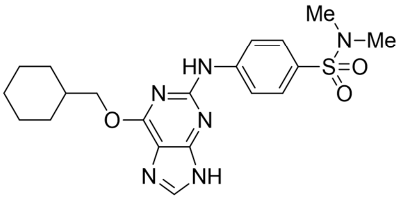

29
IC_{50}= 56 ± 20 nM

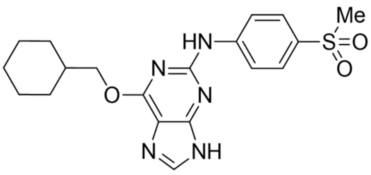

30
IC_{50}= 63 ± 7 nM

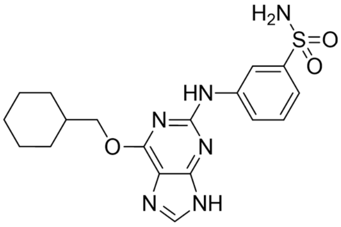

33
IC_{50}= 210 ± 40 nM

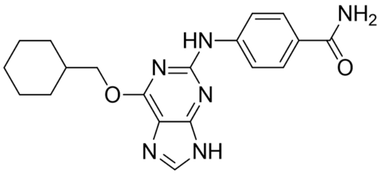

34
IC_{50}= 64 ± 33 nM

Scheme S2 Chemical structures of the seven ligands[2] docked in CDK2.

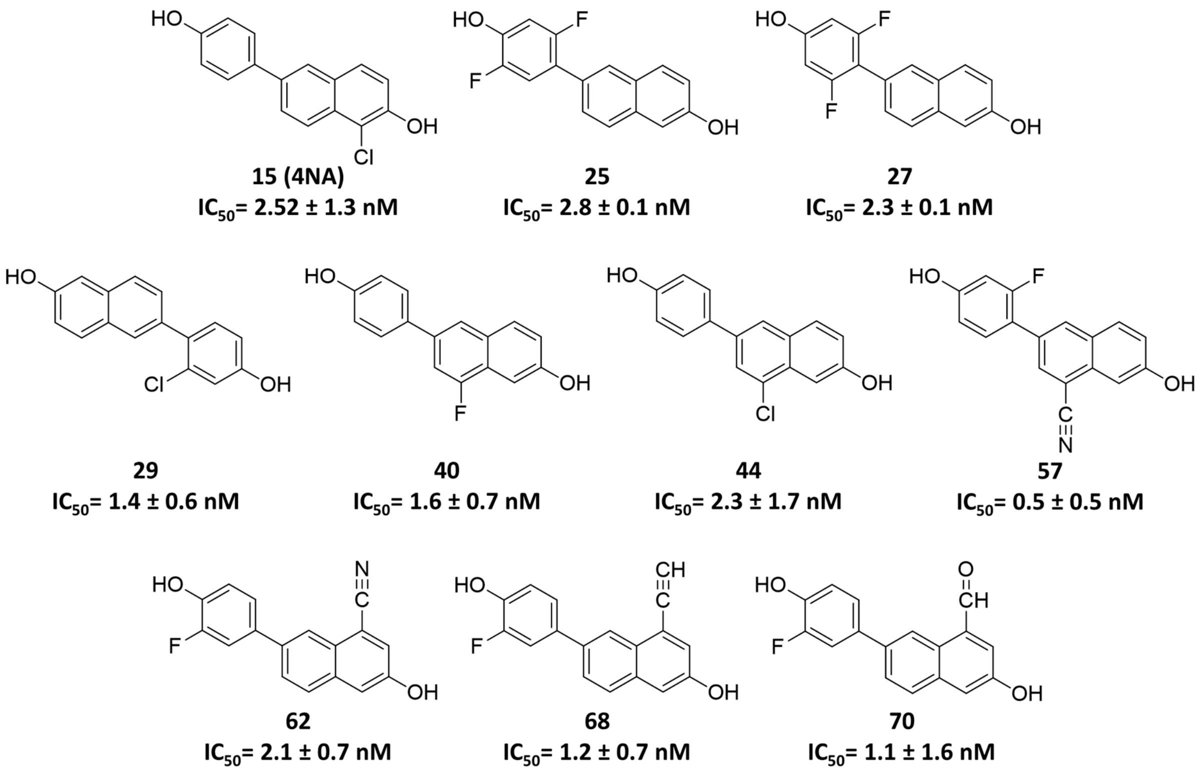

Scheme S3 Chemical structures of the ten ligands[3] docked in ERβ.

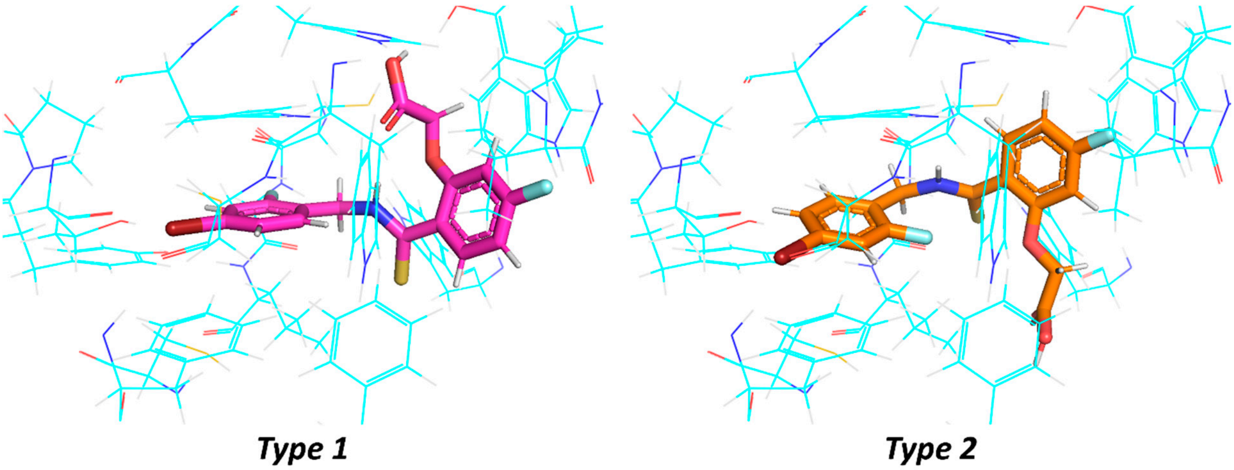

Figure S1 Two major types of orientations of the IDD594 ligand at the binding site.

Table S1 Population distribution of the ligands of hAR belonging to two major types.

Population out of 30 for ligand	Type 1	% Type 1	Type 2	% Type 2	Total of all 2 Types	% all 2 Types
**10**	21	70.00	7	23.33	28	93.33
**16**	11	36.67	11	36.67	22	73.33
**19**	11	36.67	17	56.67	28	93.33

**24**	17	56.67	11	36.67	28	93.33
**25**	10	33.33	14	46.67	24	80.00

Table S2 Interaction energies (kCal·mol^{-1}) of hAR with *GoldScore* poses of ligand 10

**Residue #**	**Type 1**			**Type 2**		
	**IE (CP)**	**IE (Raw)**	**IE (Avg)**	**IE (CP)**	**IE (Raw)**	**IE (Avg)**
20	-0.41	-6.28	-3.35	7.82	2.88	5.35
47	-2.16	-3.89	-3.03	-0.34	-0.51	-0.43
48	-20.98	-29.25	-25.12	-1.98	-4.27	-3.13
79	-3.54	-4.44	-3.99	4.26	1.27	2.77
80	-0.88	-1.04	-0.96	-0.84	-1.65	-1.25
110	-20.32	-25.15	-22.74	-2.22	-3.00	-2.61
111	-22.27	-32	-27.14	-6.24	-16.48	-11.36
113	4.24	0.26	2.25	5.22	-0.30	2.46
115	-1.65	-2.88	-2.27	-2.11	-3.13	-2.62
122	-2.53	-5.3	-3.92	3.53	-0.57	1.48
219	-1.69	-4.95	-3.32	12.73	4.16	8.45
298	-0.86	-2.12	-1.49	7.57	4.63	6.10
299	1.23	-1.38	-0.08	4.91	3.13	4.02
300	-2.32	-7.38	-4.85	-4.35	-12.52	-8.44
303	-1.63	-4.27	-2.95	-3.24	-4.02	-3.63
309	-1.4	-4.55	-2.98	2.04	-3.65	-0.81
310	-1.42	-2.04	-1.73	8.04	3.92	5.98
**Total**	-78.59	-136.66	**-107.67**	34.80	-30.11	**2.35**

Table S3 Interaction energies (kCal·mol^{-1}) of hAR with *GoldScore* poses of ligand 16

**Residue #**	**Type 1**			**Type 2**		
	**IE (CP)**	**IE (Raw)**	**IE (Avg)**	**IE (CP)**	**IE (Raw)**	**IE (Avg)**
20	-3.16	-7.3	-5.23	-2.45	-6.83	-4.64
47	-1.92	-4.71	-3.32	-1.07	-1.72	-1.40
48	-14.21	-22.97	-18.59	-0.36	-2.05	-1.21
79	-5.04	-6.82	-5.93	-0.83	-1.05	-1.40
80	-0.82	-0.87	-0.85	-0.21	-0.37	-0.29
110	-20.33	-25.75	-23.04	-0.20	-0.23	-0.22
111	-16.84	-27.65	-22.25	-2.95	-10.99	-6.97
113	1.3	0.12	0.71	-0.05	-2.96	-1.51
115	-1.52	-1.81	-1.67	-0.64	-2.43	-1.54
122	-1.58	-4.99	-3.29	-2.35	-3.66	-3.01
219	-3.47	-5.08	-4.28	5.66	1.02	3.34

298	3.07	-1.27	0.90	3.44	-0.04	1.70
299	7.62	2.51	5.07	4.15	-0.54	1.81
300	2.4	-4.97	-1.29	5.30	-2.61	1.35
303	-1.64	-2.62	-2.13	-0.55	-3.47	-2.01
309	-1.53	-4.04	-2.79	-1.48	-3.76	-2.62
310	-1.5	-2.4	-1.95	-0.44	-0.63	-0.54
**Total**	-59.17	-120.62	-89.93	4.97	-42.32	-18.68

Table S4 Interaction energies (kCal·mol^{-1}) of hAR with *GoldScore* pose of ligand 19

**Residue #**	**Type 1**			**Type 2**		
	**IE (CP)**	**IE (Raw)**	**IE (Avg)**	**IE (CP)**	**IE (Raw)**	**IE (Avg)**
20	-1.88	-8.22	-5.05	-0.19	-4.18	-2.19
47	-2.38	-3.81	-3.10	-0.31	-0.36	-0.34
48	-24.81	-32.68	-28.75	-0.10	-1.68	-0.89
79	-4.40	-5.99	-5.20	-0.61	-0.68	-0.65
80	-0.69	-1.26	-0.98	-0.23	-0.47	-0.35
110	-20.41	-25.60	-23.01	-0.30	-0.40	-0.35
111	-23.37	-36.44	-29.91	0.93	-6.33	-2.70
113	1.15	-1.64	-0.25	22.29	14.94	18.62
115	-1.95	-3.67	-2.81	1.69	-1.73	-0.02
122	-3.78	-5.66	-4.72	-1.05	-2.95	-2.00
219	-4.38	-6.96	-5.67	12.80	6.97	9.89
298	0.08	-2.41	-1.17	0.61	-3.11	-1.25
299	1.77	-2.34	-0.29	6.82	2.01	4.42
300	-2.53	-8.27	-5.40	3.02	-4.67	-0.83
303	-1.22	-2.20	-1.71	1.95	-3.23	-0.64
309	-1.56	-2.49	-2.03	-0.02	-4.08	-2.05
310	-1.37	-1.47	-1.42	-0.19	-0.67	-0.43
**Total**	**-91.73**	**-151.11**	**-121.42**	**47.11**	**10.62**	**18.25**

Table S5 Interaction energies (kCal·mol^{-1}) of hAR with *GoldScore* poses of ligand 24

**Residue #**	**Type 1**			**Type 2**		
	**IE (CP)**	**IE (Raw)**	**IE (Avg)**	**IE (CP)**	**IE (Raw)**	**IE (Avg)**
20	9.23	-0.21	4.51	-1.33	-5.67	-3.50
47	-0.41	-0.63	-0.52	-1.71	-1.82	-1.77
48	1.59	-2.83	-0.62	-22.12	-27.18	-24.65
79	-2.08	-2.67	-2.38	-4.43	-6.04	-5.24
80	-0.83	-1.01	-0.92	0.41	-0.74	-0.17
110	-2.76	-2.88	-2.82	-19.36	-23.99	-21.68
111	-5.89	-15.11	-10.50	-19.32	-31.35	-25.34
113	2	-0.43	0.79	5.51	3.41	4.46
115	-1.99	-2.67	-2.33	-1.31	-3.83	-2.57
122	-0.57	-2.81	-1.69	-3.51	-5.49	-4.50
219	-0.62	-5.08	-2.85	-2.3	-5.24	-3.77
298	-0.23	-2.24	-1.24	-0.78	-3.22	-2.00
299	1.21	-0.77	0.22	2.4	-0.85	0.78
300	-0.51	-9.57	-5.04	-3.06	-7.77	-5.42

303	-2.97	-3.86	-3.42	-1.64	-2.29	-1.97
309	-1.29	-4.96	-3.13	1.73	-1.21	0.26
310	-0.57	-2.41	-1.49	-1.1	-1.58	-1.34
**Total**	-6.69	-60.14	-33.42	-71.92	-124.86	-98.39

Table S6 Interaction energies (kCal·mol^{-1}) of hAR with *GoldScore* poses of ligand 25

**Residue**	**Type 1**			**Type 2**		
#	**IE (CP)**	**IE (Raw)**	**IE (Avg)**	**IE (CP)**	**IE (Raw)**	**IE (Avg)**
20	6.96	1.95	4.46	1.04	-4.11	-1.54
47	-0.03	-0.56	-0.30	-2.14	-2.46	-2.30
48	-1.74	-3.38	-2.56	-24.5	-30.96	-27.73
79	-1.67	-1.76	-1.72	-4.58	-6.04	-5.31
80	-0.51	-0.53	-0.52	-0.96	-1.02	-0.99
110	-0.96	-1	-0.98	-23.22	-28.01	-25.62
111	1.58	-6.59	-2.51	-15.89	-24.96	-20.43
113	-0.36	-1.25	-0.81	0.11	-1.2	-0.55
115	-1.32	-1.52	-1.42	-1.37	-1.63	-1.50
122	-0.42	-2.08	-1.25	-3.42	-4.85	-4.14
219	0.47	-4.39	-1.96	-4.24	-6.58	-5.41
298	-1.65	-3.71	-2.68	0.33	-1.53	-0.60
299	1.08	-1.42	-0.17	0.29	-2.22	-0.97
300	-4.71	-12.78	-8.75	-1.58	-5.81	-3.70
303	-3.63	-4.49	-4.06	-1.24	-1.88	-1.56
309	3.57	-2.39	0.59	1.52	-4.12	-1.30
310	6.89	2.41	4.65	5.37	0.96	3.17
**Total**	3.55	-43.49	-19.97	-74.48	-126.42	-100.45

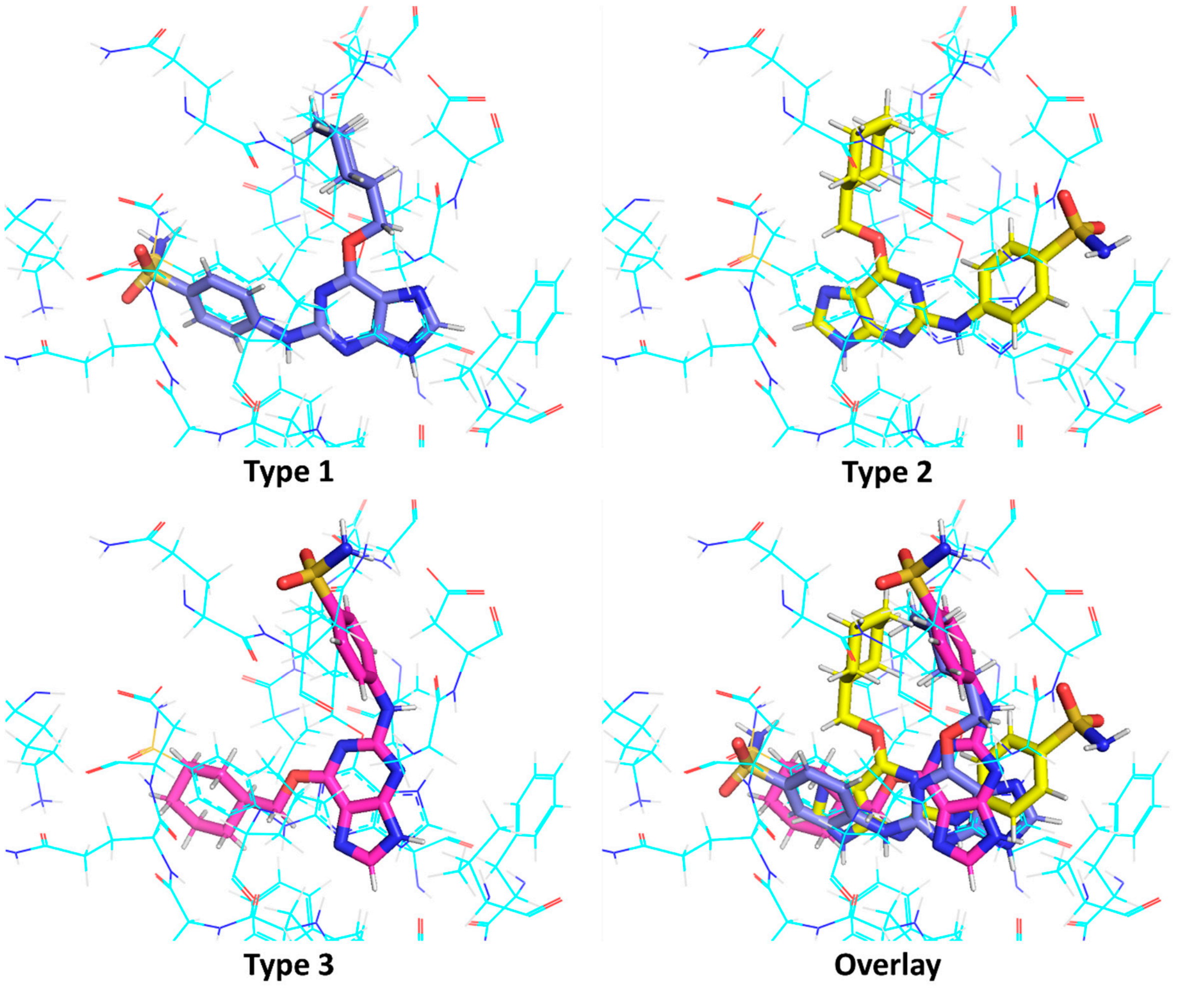

Figure S2 Three major types of orientations of the ligand NU6102 of CDK2 at the binding site and their overlay diagrams.

Table S7 Population distribution of various poses of the ligands of CDK2 belonging to three major types

Population out of 30 for ligand	Type 1	% of Type 1	Type 2	% of Type 2	Type 3	% Type 3	Total of all 3 Types	% all 3 Types
**3**	7	23.33	7	23.33	8	26.67	22	73.33
**34**	8	26.67	3	10	11	36.67	22	73.33
**25**	8	26.67	8	26.67	5	16.67	21	70.00
**28**	13	43.33	-	-	8	26.67	21	73.33
**29**	6	20	1	3.33	16	53.33	23	76.67
**30**	15	50	3	10	7	23.33	25	83.33
**33**	11	36.67	4	13.33	4	13.33	19	63.33

Table S8 Interaction energies (kCal·mol^{-1}) of CDK2 with *GoldScore* poses of ligand 3

Residue #	Crystal (NU6102)			Type 1			Type 2			Type 3		
	**IE (CP)**	**IE (Raw)**	**IE (Avg)**	**IE (CP)**	**IE (Raw)**	**IE (Avg)**	**IE (CP)**	**IE (Raw)**	**IE (Avg)**	**IE (CP)**	**IE (Raw)**	**IE (Avg)**
10	-1.83	-4.99	-3.41	-2.51	-4.7	-3.61	-1.07	-5.67	-3.37	2.81	-0.52	1.15
132	-3.44	-4.9	-4.17	-2.93	-4.28	-3.61	-2.76	-3.02	-2.89	15.64	8.55	12.10
134	-0.88	-3.46	-2.17	0.97	-1.95	-0.49	0.27	-2.13	-0.93	-2.61	-5.18	-3.90
13	1.81	0.38	1.095	3.83	1.61	2.72	1.01	0.77	0.89	1.06	4.66	2.86
144	1.13	0.97	1.05	1.15	0.78	0.97	1.24	0.25	0.75	1.55	1.22	1.39
145	-148.85	-150.57	-149.71	-147.85	-150.76	-149.31	-144.33	-147.92	-146.13	-127.11	-132.99	-130.05
146	0.62	0.62	0.62	0.66	0.66	0.66	1.93	1.61	1.77	0.72	0.72	0.72
14	-0.02	-0.03	-0.025	0.01	-0.02	-0.01	0.02	0.02	0.02	-0.09	-0.37	-0.23
18	-1.9	-3.16	-2.53	0.74	-0.76	-0.01	0.32	-1.1	-0.39	0.42	-1.13	-0.36
31	0.06	-0.9	-0.42	1.77	0.13	0.95	0.36	-0.46	-0.05	0.13	-0.52	-0.20
33	30.27	30.27	30.27	33.75	33.74	33.75	22.24	19.3	20.77	37.08	37.05	37.07
51	-144.8	-144.8	-144.8	-145.03	-145.03	-145.03	-138.71	-141.91	-140.31	-127.76	-127.76	-127.76
55	0.21	0.21	0.21	0.26	0.26	0.26	0.00	-0.19	-0.10	0.21	0.21	0.21
64	3.08	2.03	2.555	0.37	-0.26	0.06	1.66	0.06	0.86	-0.16	-0.65	-0.41
80	-2.87	-4.18	-3.525	-3.45	-5.48	-4.47	5.69	0.15	2.92	-2.71	-2.98	-2.85
81	-148.19	-150.07	-149.13	-148.39	-150.29	-149.34	-142.96	-143.54	-143.25	-89.02	-91.26	-90.14
82	-4.45	-6.72	-5.585	-4.06	-7.12	-5.59	-3.63	-6.41	-5.02	1.74	-1.32	0.21
83	-12.79	-17.3	-15.045	-9.77	-14.56	-12.17	-5.4	-9.84	-7.62	3.1	-2.24	0.43
**Total**	-432.84	-456.6	**-444.72**	-420.48	-448.03	**-434.26**	-404.12	-440.03	**-422.08**	-285	-314.51	**-299.76**

Table S9 Interaction energies (kCal·mol^{-1}) of CDK2 with *GoldScore* poses of ligand 25

Residue #	Type 1			Type 2			Type 3		
	**IE (CP)**	**IE (Raw)**	**IE (Avg)**	**IE (CP)**	**IE (Raw)**	**IE (Avg)**	**IE (CP)**	**IE (Raw)**	**IE (Avg)**
10	-2.21	-4.8	-3.51	1.6	-1.85	-0.13	0.47	-4.42	-1.98
132	-2.77	-3.98	-3.38	-2.86	-3.5	-3.18	-2.04	-2.04	-2.04
134	-0.15	-2.66	-1.41	3.27	-0.17	1.55	-1.11	-3.06	-2.09
13	3.65	1.31	2.48	1.85	0.96	1.41	2.19	2.18	2.19
144	0.72	0.4	0.56	1.75	0.95	1.35	3.04	1.61	2.33
145	-125.39	-128.16	-126.78	-134.71	-136.48	-135.60	-120.02	-123.53	-121.78
146	0.5	0.49	0.50	1.8	1.67	1.74	2.07	1.07	1.57
14	-0.06	-0.08	-0.07	0.38	0.38	0.38	0.63	0.63	0.63
18	0.38	-1.34	-0.48	-0.34	-1.69	-1.02	-0.06	-1.46	-0.76
31	1.32	-0.18	0.57	0	-0.97	-0.49	1.31	0.03	0.67
33	27.86	27.84	27.85	25.71	21.98	23.85	55.99	54.92	55.46
51	-127.8	-127.8	-127.80	-103.11	-98.89	-101.00	-104.44	-109.44	-106.94
55	0.2	0.2	0.20	0.25	0.17	0.21	0.34	-0.29	0.03
64	0.7	0.02	0.36	0.96	-0.81	0.08	2.91	1.06	1.99
80	-2.65	-4.02	-3.34	-0.5	-4.82	-2.66	-1.47	-3.9	-2.69
81	-52.79	-54.98	-53.89	-40.57	-40.75	-40.66	-41.59	-41.95	-41.77
82	-4.82	-8.04	-6.43	-0.13	-2.22	-1.18	-0.13	-2.22	-1.18
83	-8.03	-12.81	-10.42	-1.41	-4.42	-2.92	-1.41	-4.42	-2.92
**Total**	**-291.34**	**-318.59**	**-304.97**	**-246.06**	**-270.46**	**-258.26**	**-203.32**	**-235.23**	**-219.28**

Table S10 Interaction energies (kCal·mol^{-1}) of CDK2 with *GoldScore* poses of ligand 28

Residue #	Type 1			Type 3		
	**IE (CP)**	**IE (Raw)**	**IE (Avg)**	**IE (CP)**	**IE (Raw)**	**IE (Avg)**
10	-3.72	-6.63	-5.18	2.5	-1.93	0.29
132	-3.11	-4.34	-3.73	0.94	-4.03	-1.55
134	1.99	-1.2	0.40	0.26	-1.27	-0.51
13	6.6	3.84	5.22	3.46	0.53	2.00
144	1.2	0.9	1.05	1.16	1.14	1.15
145	-147.48	-150.09	-148.79	-123.68	-128.78	-126.23
146	0.69	0.69	0.69	0.13	0.13	0.13
14	-0.01	-0.04	-0.03	-0.61	-2.55	-1.58
18	0	-1.45	-0.73	-0.87	-2.95	-1.91
31	0.17	-0.93	-0.38	0.16	-0.59	-0.22
33	33.85	33.84	33.85	41.68	41.68	41.68
51	-144.55	-144.55	-144.55	-124.51	-124.51	-124.51
55	0.27	0.27	0.27	0.52	0.52	0.52
64	0.47	-0.25	0.11	0.72	0.71	0.72
80	-4.34	-5.97	-5.16	-0.53	-0.55	-0.54
81	-148.13	-149.59	-148.86	-123.85	-124.17	-124.01
82	-3.59	-6.08	-4.84	0.53	0.15	0.34
83	-10.43	-15.64	-13.04	0.11	-0.94	-0.42
**Total**	**-420.12**	**-447.22**	**-433.67**	**-321.88**	**-347.41**	**-334.65**

Table S11 Interaction energies (kCal·mol^{-1}) of CDK2 with *GoldScore* poses of ligand 29

Residue #	Type 1			Type 2			Type 3		
	IE (CP)	IE (Raw)	IE (Avg)	IE (CP)	IE (Raw)	IE (Avg)	IE (CP)	IE (Raw)	IE (Avg)
10	-3.22	-6.32	-4.77	-3.07	-4.97	-4.02	6.69	3.86	5.28
132	-3.04	-4.21	-3.63	-3.81	-4.85	-4.33	-6.45	-7.83	-7.14
134	0.72	-2.08	-0.68	2.81	-0.26	1.28	4.06	1.47	2.77
13	6.08	3.44	4.76	1.21	0.79	1.00	8.87	5.29	7.08
144	1.21	0.95	1.08	1.85	1.08	1.47	1.03	0.68	0.86
145	-147.87	-150.14	-149.01	-144.98	-146.39	-145.69	-139.81	-145.86	-142.84
146	0.68	0.68	0.68	0.73	0.69	0.71	0.69	0.69	0.69
14	0	-0.03	-0.02	0.11	0.11	0.11	0.14	0	0.07
18	-0.05	-1.61	-0.83	1.26	-1.76	-0.25	-1.08	-2.27	-1.68
31	1.39	-0.17	0.61	0.67	-0.83	-0.08	-0.62	-1.35	-0.99
33	33.71	33.7	33.71	27.76	26.96	27.36	46.19	45.84	46.02
51	-144.74	-144.74	-144.74	-141.91	-142.33	-142.12	-139.09	-139.11	-139.10
55	0.27	0.27	0.27	0.22	0.19	0.21	0.49	0.49	0.49
64	0.2	-0.32	-0.06	1.55	0.06	0.81	-0.15	-0.53	-0.34
80	-4.25	-6.15	-5.20	15.67	8.33	12.00	-6.48	-8.32	-7.40
81	-148.13	-149.56	-148.85	-118.55	-119.05	-118.80	-143.23	-143.27	-143.25
82	-3.34	-6.17	-4.76	-1.03	-1.49	-1.26	0.5	-1.55	-0.53
83	-10.26	-15.12	-12.69	-3.86	-6.69	-5.28	5.43	1.45	3.44
**Total**	**-420.64**	**-447.58**	**-434.11**	**-363.37**	**-390.41**	**-376.89**	**-362.82**	**-390.32**	**-376.57**

Table S12 Interaction energies (kCal·mol^{-1}) of CDK2 with *GoldScore* poses of ligand 30

Residue #	Type 1			Type 2			Type 3		
	IE (CP)	IE (Raw)	IE (Avg)	IE (CP)	IE (Raw)	IE (Avg)	IE (CP)	IE (Raw)	IE (Avg)
10	-0.78	-3.78	-2.28	-3.48	-7.23	-5.36	0.38	-2.65	-1.14
132	-2.67	-3.14	-2.91	-3.41	-4.34	-3.88	-5.12	-6.85	-5.99
134	0.74	-1.83	-0.55	0.02	-2.42	-1.20	-0.82	-3.46	-2.14
13	7.15	4.5	5.83	1.62	0.86	1.24	0.9	-0.82	0.04
144	1.02	0.67	0.85	1.25	1.01	1.13	1.51	1.31	1.41
145	-145.88	-149.5	-147.69	-148.95	-149.42	-149.19	-123.74	-124.27	-124.01
146	0.63	0.63	0.63	-20.58	-21.19	-20.89	0.85	0.85	0.85
14	-0.05	-0.12	-0.09	0.12	0.12	0.12	0.17	0.15	0.16
18	-1.36	-2.44	-1.90	-0.6	-2.79	-1.70	-0.07	-1.78	-0.93
31	2.08	0.48	1.28	0.79	-0.71	0.04	0.25	-0.35	-0.05
33	30.38	30.37	30.38	5.27	4.12	4.70	38.66	38.65	38.66
51	-144.21	-144.21	-144.21	-145.74	-146.08	-145.91	-119.4	-119.4	-119.40
55	0.2	0.2	0.20	0.21	0.21	0.21	0.21	0.21	0.21
64	0.93	0.24	0.59	0.18	-1.38	-0.60	0.1	-0.27	-0.09
80	-3.3	-4.38	-3.84	-1.1	-3.91	-2.51	-15.95	-16.17	-16.06

81	-143.03	-145.91	-144.47	-143.98	-144.78	-144.38	-119.67	-121.62	-120.65
82	9.96	4.39	7.18	-26.56	-28.95	-27.76	-12.16	-14.61	-13.39
83	5.87	-0.91	2.48	-0.23	-4.64	-2.44	2.23	-2.47	-0.12
**Total**	**-382.32**	**-414.74**	**-398.53**	**-485.17**	**-511.52**	**-498.35**	**-351.67**	**-373.55**	**-362.61**

Table S13 Interaction energies (kCal·mol^{-1}) of CDK2 with *GoldScore* poses of ligand 33

**Residue** #	**Type 1**			**Type 2**			**Type 3**		
	**IE (CP)**	**IE (Raw)**	**IE (Avg)**	**IE (CP)**	**IE (Raw)**	**IE (Avg)**	**IE (CP)**	**IE (Raw)**	**IE (Avg)**
10	-1.78	-5.62	-3.70	0.80	-2.77	-0.99	8.38	5.30	6.84
132	-3.23	-4.30	-3.77	-3.73	-4.11	-3.92	16.01	11.40	13.71
134	-0.30	-2.87	-1.59	0.34	-2.13	-0.90	2.90	0.44	1.67
13	4.58	2.11	3.35	0.48	-0.36	0.06	-10.93	-15.64	-13.29
144	1.24	0.95	1.10	1.56	0.96	1.26	1.13	0.66	0.90
145	-146.86	-149.19	-148.03	-113.71	-121.79	-117.75	-75.45	-82.67	-79.06
146	0.68	0.68	0.68	1.52	1.35	1.44	0.65	0.65	0.65
14	0.11	-0.02	-0.01	-0.35	-0.59	-0.47	0.33	-0.54	-0.11
18	-0.10	-1.67	-0.89	5.37	2.50	3.94	-1.03	-2.17	-1.60
31	0.93	-0.46	0.24	1.09	-0.34	0.38	-0.55	-1.21	-0.88
33	33.57	33.56	33.57	-0.18	-3.64	-1.91	48.71	48.43	48.57
51	-143.80	-143.80	-143.80	-135.45	-138.14	-136.80	-138.56	-138.57	-138.57
55	0.26	0.26	0.26	0.09	-0.01	0.04	0.51	0.50	0.51
64	0.62	-0.03	0.30	1.02	-0.04	0.49	0.02	-0.59	-0.29
80	-3.96	-5.33	-4.65	-0.36	-4.18	-2.27	-16.24	-17.73	-16.99
81	-147.69	-149.55	-148.62	-140.63	-140.78	-140.71	-142.74	-142.81	-142.78
82	-5.02	-8.42	-6.72	-4.41	-6.83	-5.62	0.86	-1.32	-0.23
83	-8.35	-12.85	-10.60	-8.54	-12.14	-10.34	5.73	1.73	3.73
**Total**	-419.21	-446.55	**-432.88**	-395.09	-433.04	**-414.07**	-300.27	-334.14	**-317.21**

Table S14 Interaction energies (kCal·mol^{-1}) of CDK2 with *GoldScore* poses of ligand 34

**Residue**	**Type 1**			**Type 2**		
#	**IE (CP)**	**IE (Raw)**	**IE (Avg)**	**IE (CP)**	**IE (Raw)**	**IE (Avg)**
10	-1.42	-3.62	-2.52	1.29	-2.76	-0.74
132	-2.19	-3.8	-3.00	-3.65	-4.94	-4.30
134	0.72	-2.16	-0.72	0.91	-1.67	-0.38
13	3.23	1.27	2.25	2.49	1.51	2.00
144	1.01	0.68	0.85	3.24	1.50	2.37
145	-141.69	-144.2	-142.95	-123.20	-126.75	-124.98
146	0.63	0.63	0.63	-1.32	-2.76	-2.04
14	0.02	0.00	0.01	-0.02	-0.03	-0.03
18	-0.52	-1.81	-1.17	-0.97	-2.05	-1.51
31	1.59	0.05	0.82	2.82	1.01	1.92
33	31.2	31.19	31.20	36.69	35.71	36.20
51	-138.56	-138.56	-138.56	-104.57	-109.47	-107.02
55	0.23	0.23	0.23	0.15	-0.97	-0.41

64	0.34	-0.21	0.07	11.35	7.13	9.24
80	-3.95	-5.56	-4.76	0.88	-4.84	-1.98
81	-142.13	-143.76	-142.95	-118.88	-119.24	-119.06
82	-3.88	-6.78	-5.33	-5.74	-7.60	-6.67
83	-9.8	-14.41	-12.11	-15.37	-18.12	-16.75
**Total**	**-405.17**	**-430.82**	**-418.00**	**-313.9**	**-354.34**	**-334.12**

Table S15 Comparison of ranking based on the experimental IC_{50} values, the average IE from KEM-CP and fitting score from *GoldScore* of the best poses of the CDK2 ligands.

**Best Poses**						
**Ligand #**	**Expt. IC_{50}**		**KEM-CP**		**GoldScore**	
	**Value (nM)**	**Ranking**	**IE (kCal·mol^{-1})**	**Ranking**	**Fitness Score**	**Ranking**
3	5.4 ± 1.0	**1**	-434.26	**2**	67.87	**3**
25	69 ± 1	**6**	-304.97	**7**	62.98	**7**
28	7.0 ± 0.1	**2**	-433.67	**4**	68.88	**2**
29	56 ± 20	**3**	-434.11	**3**	73.24	**1**
30	63 ± 7	**4**	-498.35	**1**	66.33	**5**
33	210 ± 40	**7**	-432.88	**5**	66.71	**4**
34	64 ± 33	**5**	-418.00	**6**	64.60	**6**
**Correlation**				***0.64***		***0.64***

Table S16 Comparison of ranking based on the experimental IC_{50} values, the average IE from KEM-CP and fitting score from *GoldScore* of the Type 1 poses of the CDK2 ligands.

**Pose Type 1**						
**Ligand #**	**Expt IC_{50}**		**KEM-CP**		**GoldScore**	
	**Value (nM)**	**Ranking**	**IE (kCal·mol^{-1})**	**Ranking**	**Fitness Score**	**Ranking**
3	5.4 ± 1.0	**1**	-434.26	**1**	67.87	**3**
25	69 ± 1	**6**	-304.97	**7**	62.98	**7**
28	7.0 ± 0.1	**2**	-433.67	**3**	68.88	**2**
29	56 ± 20	**3**	-434.11	**2**	73.24	**1**
30	63 ± 7	**4**	-398.53	**6**	66.33	**5**
33	210 ± 40	**7**	-432.88	**4**	66.71	**4**
34	64 ± 33	**5**	-418.00	**5**	64.60	**6**
**Correlation**				***0.71***		***0.64***

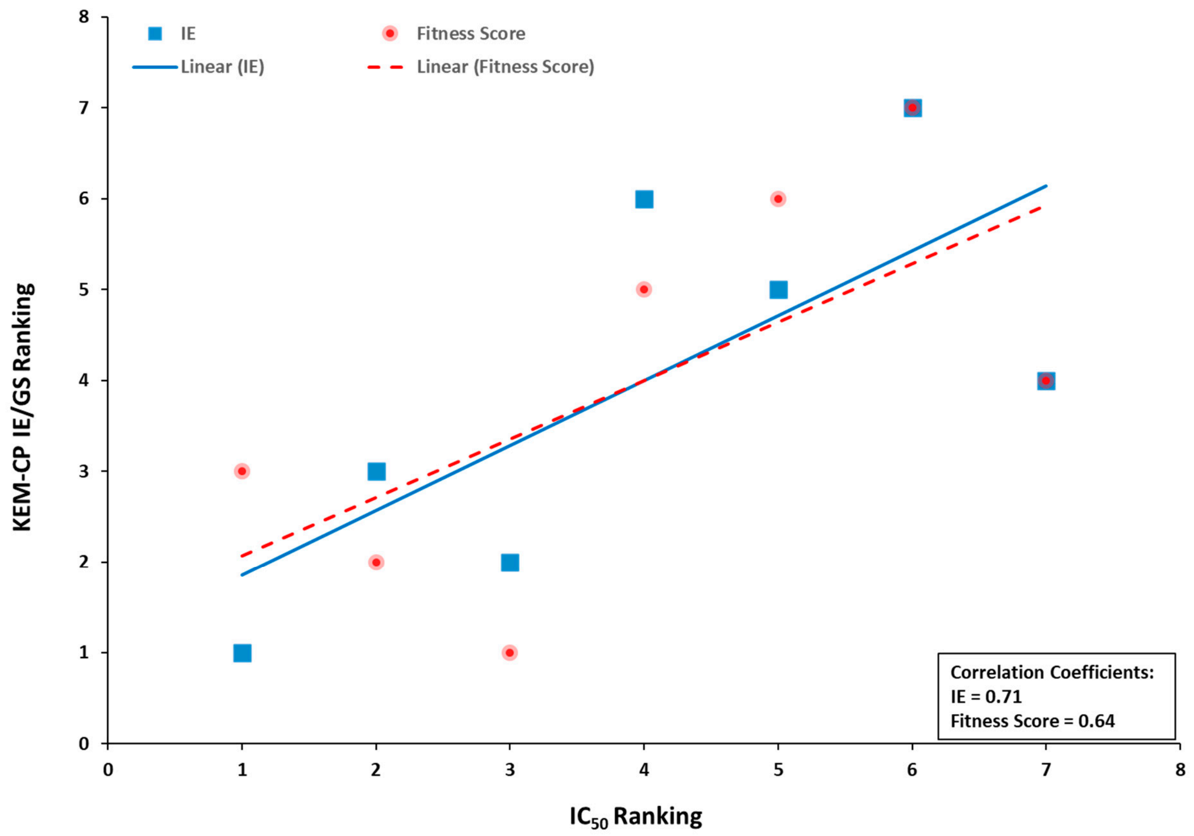

Figure S3 Plot showing IC_{50} ranking distribution with the KEM-CP IE and *GoldScore* Fitness score rankings for pose Type 1 (from **Table S16**)

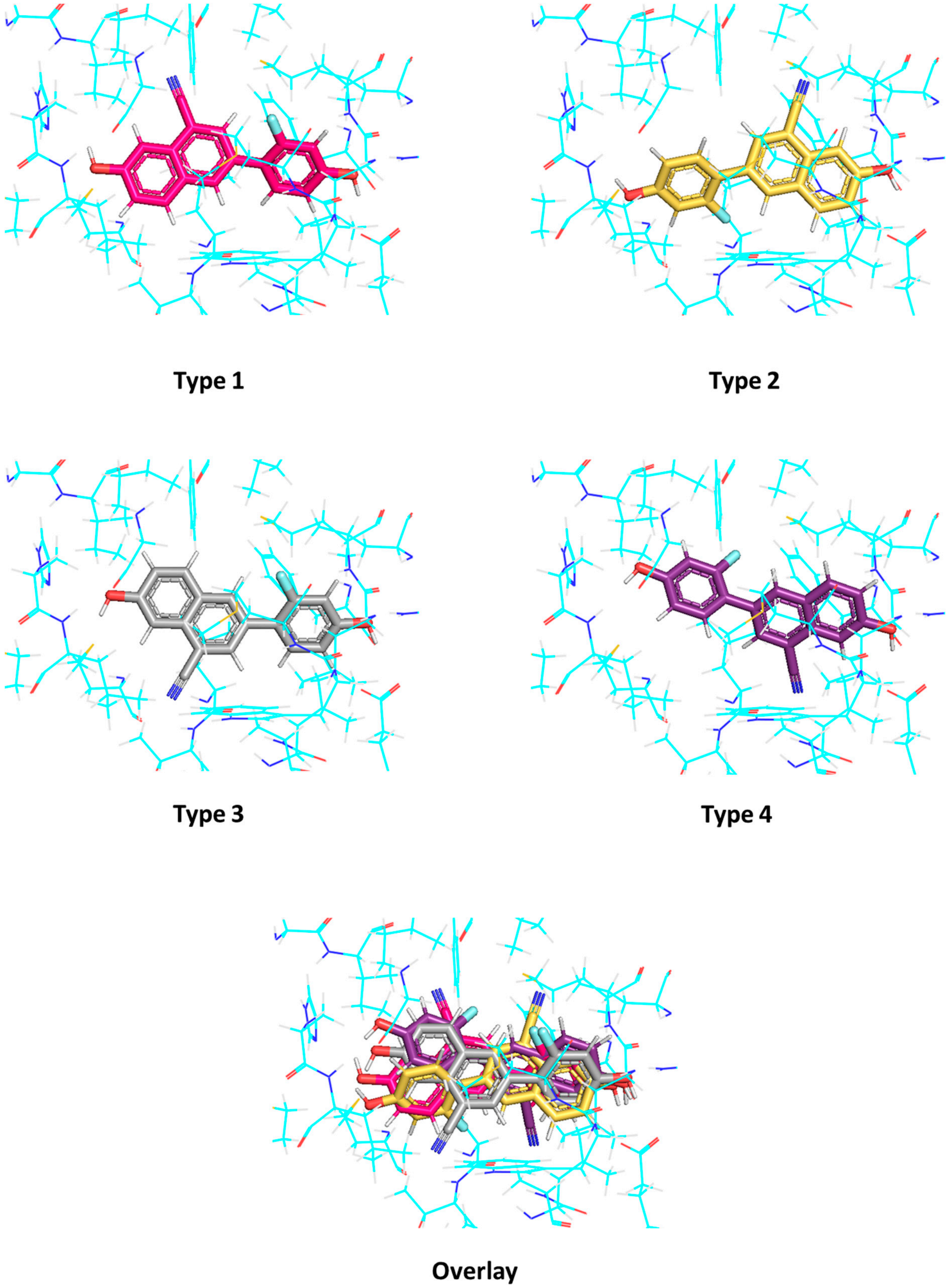

Figure S4 Four major types of orientations of the ligand 4NA of ERβ at the binding site and their overlay diagrams.

Table S17 Population distribution of the 30 poses of the ligands of ERβ belonging to 4 types

**Population out of 30 for**	**Type 1**	**% Type 1**	**Type 2**	**% Type 2**	**Type 3**	**% Type 3**	**Type 4**	**% Type 4**
**15**	10	33.33	9	30.00	5	16.67	6	20.00
**25**	5	16.67	8	26.67	9	30.00	8	26.67
**27**	6	20.00	7	23.33	10	33.33	7	23.33
**29**	7	23.33	7	23.33	10	33.33	6	20.00
**40**	10	33.33	7	23.33	7	23.33	6	20.00
**44**	10	33.33	9	30.00	5	16.67	6	20.00
**57**	11	36.67	7	23.33	3	10.00	9	30.00
**62**	7	23.33	10	33.33	7	23.33	6	20.00
**68**	11	36.67	8	26.67	4	13.33	7	23.33
**70**	8	26.67	10	33.33	7	23.33	5	16.67

Table S18 Interaction energies (kCal·mol^{-1}) of ERβ with *GoldScore* poses of ligand 15

Residue #	Crystal (4NA)			Type 1			Type 2			Type 3			Type 4		
	**IE (CP)**	**IE (Raw)**	**IE (Avg)**	**IE (CP)**	**IE (Raw)**	**IE (Avg)**	**IE (CP)**	**IE (Raw)**	**IE (Avg)**	**IE (CP)**	**IE (Raw)**	**IE (Avg)**	**IE (CP)**	**IE (Raw)**	**IE (Avg)**
295	-0.1	1.93	0.92	-1.60	0.19	-0.71	-1.77	-0.08	-0.93	1.98	3.27	2.63	-0.15	1.06	0.46
298	-7.65	-4.52	-6.09	-3.02	0.65	-1.19	-6.13	-2.53	-4.33	-4.81	-0.96	-2.89	-6.28	-2.42	-4.35
299	-1.24	-0.73	-0.99	-0.92	0.40	-0.26	-0.78	-0.56	-0.67	-1.61	-0.72	-1.17	-0.59	-0.35	-0.47
301	-1.51	-0.22	-0.87	-1.33	-1.03	-1.18	-1.29	0.17	-0.56	-1.61	-1.20	-1.41	1.33	3.21	2.27
302	-1.52	-0.53	-1.03	-1.13	-0.23	-0.68	-1.61	-0.09	-0.85	-1.63	-0.39	-1.01	-1.16	1.33	0.09
305	-31.35	-23.7	-27.53	-27.91	-22.48	-25.20	-30.47	-25.87	-28.17	-26.35	-20.75	-23.55	-8.63	-2.26	-5.45
336	-2.32	-1.65	-1.99	-3.08	-0.87	-1.98	-2.09	-0.71	-1.40	-4.15	-2.42	-3.29	-3.01	-2.03	-2.52
339	-2.93	1.37	-0.78	0.44	7.79	4.12	-1.21	2.63	0.71	1.89	8.77	5.33	-4.36	-1.59	-2.98
340	-1.96	-0.65	-1.31	-4.03	-2.16	-3.10	0.82	4.38	2.60	-3.93	-2.34	-3.14	-2.41	-0.71	-1.56
343	-2.62	-1.66	-2.14	-1.85	0.23	-0.81	-2.38	-1.03	-1.71	-2.07	0.35	-0.86	-3.05	-0.53	-1.79
346	-7.84	-6.48	-7.16	-7.38	-5.78	-6.58	-6.11	-5.63	-5.87	-6.10	-4.28	-5.19	-6.08	-5.11	-5.60
356	-5.59	-3.83	-4.71	-3.16	-2.40	-2.78	-4.90	-3.47	-4.19	-3.54	-2.61	-3.08	-4.90	-3.17	-4.04
373	1.51	3.25	2.38	-0.46	-0.36	-0.41	-0.89	0.14	-0.38	-0.77	-0.59	-0.68	-1.23	-0.71	-0.97
376	2.47	4.03	3.25	-0.75	-0.55	-0.65	-1.04	-0.61	-0.83	-0.98	-0.74	-0.86	-0.96	-0.74	-0.85
377	-0.61	-0.56	-0.59	-0.25	-0.25	-0.25	-0.22	-0.21	-0.22	-0.25	-0.25	-0.25	-0.29	-0.29	-0.29
380	-0.59	-0.48	-0.54	-0.34	-0.31	-0.33	-0.55	-0.46	-0.51	-0.29	-0.26	-0.28	-0.52	-0.38	-0.45
472	-0.78	-0.15	-0.47	6.17	9.34	7.76	-0.85	-0.23	-0.54	0.31	3.17	1.74	0.58	3.18	1.88
475	-1.16	2.37	0.61	-4.17	-2.45	-3.31	15.64	22.13	18.89	-3.48	-1.24	-2.36	-0.49	5.35	2.43
476	-5.82	-2.69	-4.26	-4.38	0.48	-1.95	-3.90	1.40	-1.25	-3.40	1.06	-1.17	-3.47	0.97	-1.25
479	-0.31	-0.06	-0.19	-0.86	-0.61	-0.74	-0.53	0.12	-0.21	-0.34	-0.27	-0.31	-0.41	-0.20	-0.31
491	-0.06	-0.05	-0.06	-0.13	-0.12	-0.13	-0.05	-0.05	-0.05	-0.13	-0.13	-0.13	-0.13	-0.13	-0.13
**Total**	-71.98	-35.01	**-53.50**	-60.14	-20.52	**-40.33**	-50.31	-10.56	**-30.44**	-61.26	-22.53	**-41.90**	-46.21	-5.52	**-25.87**

Table S19 Interaction energies (kCal·mol^{-1}) of ERβ with *GoldScore* poses of ligand 25

Residue #	Type 1			Type 2			Type 3			Type 4		
	**IE (CP)**	**IE (Raw)**	**IE (Avg)**	**IE (CP)**	**IE (Raw)**	**IE (Avg)**	**IE (CP)**	**IE (Raw)**	**IE (Avg)**	**IE (CP)**	**IE (Raw)**	**IE (Avg)**
295	-1.98	0.12	-0.93	-2.66	-0.64	-1.65	-0.39	-0.11	-0.25	-4.53	-1.72	-3.13
298	-7.29	-2.86	-5.08	-3.44	-0.94	-2.19	-3.42	-0.66	-2.04	-3.61	2.69	-0.46
299	-1.49	-0.99	-1.24	-0.86	-0.78	-0.82	-0.89	-0.74	-0.82	-2.67	-1.04	-1.86
301	-2.16	-1.03	-1.60	-1.59	-1.19	-1.39	-2.45	-1.14	-1.80	-2.59	-1.56	-2.08
302	-1.99	-0.92	-1.46	-1.06	-0.29	-0.68	-2.24	0.23	-1.01	-2.61	-0.41	-1.51
305	-31.78	-26.68	-29.23	-28.67	-22.82	-25.75	-33.90	-28.14	-31.02	-30.46	-24.22	-27.34
336	-1.39	-1.00	-1.20	-2.05	0.52	-0.77	-2.68	-1.66	-2.17	-1.30	-0.99	-1.15
339	-1.01	4.31	1.65	-0.43	6.28	2.93	-2.65	1.57	-0.54	3.62	10.24	6.93
340	-3.60	0.27	-1.67	-2.10	0.31	-0.90	-4.40	-1.31	-2.86	-0.76	1.75	0.50
343	-4.14	-1.44	-2.79	-2.53	0.09	-1.22	-3.20	-0.09	-1.65	-2.70	0.10	-1.30
346	-7.49	-6.41	-6.95	-8.50	-6.63	-7.57	-7.92	-6.58	-7.25	-9.04	-6.95	-8.00
356	-4.81	-2.59	-3.70	-4.44	-3.11	-3.78	-4.80	-3.29	-4.05	-5.99	-3.86	-4.93
373	-0.65	0.29	-0.18	-1.00	-0.60	-0.80	-1.07	0.60	-0.24	-0.42	0.65	0.12
376	-0.71	-0.47	-0.59	-0.82	-0.67	-0.75	-1.02	-0.11	-0.57	-0.17	-0.13	-0.15
377	-0.44	-0.30	-0.37	-0.28	-0.27	-0.28	-0.42	-0.32	-0.37	-0.85	-0.43	-0.64
380	-0.53	-0.39	-0.46	-0.90	-0.59	-0.75	-0.45	-0.37	-0.41	-0.35	-0.30	-0.33
472	-0.31	-0.25	-0.28	-6.21	-3.07	-4.64	-1.67	-1.01	-1.34	-0.02	-0.02	-0.02
475	-6.80	-3.92	-5.36	-2.56	-0.85	-1.71	-0.99	3.69	1.35	-2.11	-1.23	-1.67
476	-3.59	-0.56	-2.08	-3.21	-0.14	-1.68	-2.65	0.59	-1.03	-1.57	0.76	-0.41
479	-1.33	-0.85	-1.09	-0.19	-0.13	-0.16	0.22	0.28	0.25	-0.72	-0.64	-0.68
491	-0.11	-0.10	-0.11	-0.08	-0.08	-0.08	-0.19	-0.17	-0.18	-0.11	-0.11	-0.11
**Total**	**-83.60**	**-45.77**	**-64.69**	**-73.58**	**-35.60**	**-54.59**	**-77.18**	**-38.74**	**-57.96**	**-68.96**	**-27.42**	**-48.19**

Table S20 Interaction energies (kCal·mol^{-1}) of ERβ with *GoldScore* poses of ligand 27

**Residue #**	**Type 1**			**Type 2**			**Type 3**			**Type 4**		
	**IE (CP)**	**IE (Raw)**	**IE (Avg)**	**IE (CP)**	**IE (Raw)**	**IE (Avg)**	**IE (CP)**	**IE (Raw)**	**IE (Avg)**	**IE (CP)**	**IE (Raw)**	**IE (Avg)**
295	-1.63	-0.76	-1.20	-0.79	1.27	0.24	-0.72	-0.39	-0.56	-1.51	0.23	-0.64
298	-2.57	0.05	-1.26	-5.57	-2.46	-4.02	-3.05	0.84	-1.11	-5.14	0.24	-2.45
299	-1.25	-0.69	-0.97	-2.03	-1.31	-1.67	-0.91	-0.48	-0.70	-1.96	-1.25	-1.61
301	-1.47	-1.18	-1.33	-1.48	-0.54	-1.01	-1.98	-1.43	-1.71	-2.53	-1.64	-2.09
302	-0.35	2.05	0.85	-1.92	-0.87	-1.40	1.42	5.06	3.24	-2.87	-0.40	-1.64
305	-31.51	-25.84	-28.68	-22.74	-18.98	-20.86	-29.36	-23.66	-26.51	-30.00	-23.71	-26.86
336	-2.48	-0.16	-1.32	-2.37	-0.14	-1.26	-4.13	-2.44	-3.29	-1.30	-0.91	-1.11
339	-3.32	2.89	-0.22	0.00	0.00	0.00	0.20	6.93	3.57	3.31	10.17	6.74
340	-5.16	-2.60	-3.88	-2.96	-1.53	-2.25	-4.11	-2.05	-3.08	-1.23	1.29	0.03
343	4.51	8.06	6.29	-2.37	-1.61	-1.99	-2.01	0.53	-0.74	-2.14	0.65	-0.75
346	-6.28	-4.43	-5.36	-3.74	-3.30	-3.52	-4.28	-2.59	-3.44	-10.88	-8.82	-9.85
356	-4.43	-2.85	-3.64	-4.21	-2.99	-3.60	-4.50	-2.89	-3.70	-5.93	-3.83	-4.88
373	-0.45	-0.29	-0.37	-0.43	0.40	-0.02	-0.69	-0.46	-0.58	-0.95	0.45	-0.25
376	-0.52	-0.42	-0.47	-1.51	-0.67	-1.09	-0.99	-0.73	-0.86	-0.42	-0.38	-0.40
377	-0.28	-0.28	-0.28	-0.35	-0.34	-0.35	-0.34	-0.34	-0.34	-1.11	-0.61	-0.86
380	-0.71	-0.55	-0.63	-0.41	-0.37	-0.39	-0.54	-0.47	-0.51	-0.45	-0.40	-0.43
472	-3.73	-2.16	-2.95	-1.75	-0.94	-1.35	0.58	3.43	2.01	-0.59	-0.59	-0.59
475	-2.15	-0.66	-1.41	-12.67	-6.78	-9.73	-4.03	-1.51	-2.77	-2.83	-1.83	-2.33
476	-4.53	-0.40	-2.47	-3.62	1.94	-0.84	-4.49	-0.48	-2.49	-1.70	-0.66	-1.18
479	-0.53	-0.43	-0.48	-1.77	-0.57	-1.17	-0.17	-0.11	-0.14	0.31	0.37	0.34
491	-0.15	-0.15	-0.15	-0.18	-0.18	-0.18	-0.12	-0.11	-0.12	-0.22	-0.21	-0.22
**Total**	**-68.99**	**-30.80**	**-49.90**	**-72.87**	**-39.97**	**-56.42**	**-64.22**	**-23.35**	**-43.79**	**-70.14**	**-31.84**	**-50.99**

Table S21 Interaction energies (kCal·mol^{-1}) of ERβ with *GoldScore* poses of ligand 29

**Residue #**	**Type 1**			**Type 2**			**Type 3**			**Type 4**		
	**IE (CP)**	**IE (Raw)**	**IE (Avg)**	**IE (CP)**	**IE (Raw)**	**IE (Avg)**	**IE (CP)**	**IE (Raw)**	**IE (Avg)**	**IE (CP)**	**IE (Raw)**	**IE (Avg)**
295	-0.79	1.68	0.45	1.34	3.77	2.56	-1.40	0.95	-0.23	-1.61	-0.36	-0.99
298	-5.98	-0.37	-3.18	-3.55	0.84	-1.36	-3.58	-1.22	-2.40	-6.21	-1.39	-3.80
299	-1.95	-1.07	-1.51	-2.44	-0.63	-1.54	-1.23	-1.08	-1.16	-1.08	-0.73	-0.91
301	-1.27	0.62	-0.33	-1.33	-0.18	-0.76	-1.77	-1.36	-1.57	-2.52	-1.67	-2.10
302	-2.31	-1.16	-1.74	-2.40	-1.20	-1.80	-1.34	0.08	-0.63	-2.36	0.11	-1.13
305	-30.37	-24.86	-27.62	-4.33	-0.53	-2.43	-29.42	-23.56	-26.49	-30.44	-24.17	-27.31
336	-1.98	-1.60	-1.79	-2.47	0.22	-1.13	-1.68	0.15	-0.77	-1.37	-0.81	-1.09
339	-1.90	2.35	0.23	-8.66	-3.65	-6.16	-2.15	4.35	1.10	2.88	9.76	6.32
340	-1.12	0.64	-0.24	-1.25	0.89	-0.18	0.12	2.59	1.36	-1.28	1.22	-0.03
343	-3.78	-2.09	-2.94	-2.87	-0.89	-1.88	-2.60	-0.38	-1.49	-1.77	1.10	-0.34
346	-7.93	-6.21	-7.07	-7.80	-6.54	-7.17	-5.36	-3.71	-4.54	-10.84	-8.76	-9.80
356	-4.86	-1.86	-3.36	-3.88	-2.05	-2.97	-3.88	-2.50	-3.19	-4.44	-2.16	-3.30
373	-0.85	-0.25	-0.55	-0.56	-0.14	-0.35	0.40	1.57	0.99	-1.28	-0.08	-0.68
376	-0.51	-0.37	-0.44	-0.40	-0.29	-0.35	-0.37	-0.36	-0.37	-0.64	-0.55	-0.60
377	-0.46	-0.34	-0.40	-0.13	-0.13	-0.13	-0.47	-0.38	-0.43	-1.37	-0.81	-1.09
380	-0.48	-0.35	-0.42	-0.20	-0.19	-0.20	-0.93	-0.65	-0.79	-0.72	-0.57	-0.65
472	-0.24	-0.22	-0.23	-0.74	-0.49	-0.62	-0.81	-0.79	-0.80	-0.83	-0.82	-0.83
475	-5.60	-4.11	-4.86	-8.68	-6.76	-7.72	-1.28	-0.28	-0.78	-2.61	-1.49	-2.05
476	-2.62	-0.26	-1.44	-3.41	-0.34	-1.88	-1.37	1.21	-0.08	-1.29	0.16	-0.57
479	-1.02	-0.68	-0.85	-1.02	-0.54	-0.78	-0.23	0.00	-0.12	0.26	0.32	0.29
491	-0.19	-0.17	-0.18	-0.63	-0.49	-0.56	-0.32	-0.26	-0.29	-0.13	-0.12	-0.13
**Total**	**-76.21**	**-40.68**	**-58.45**	**-55.41**	**-19.32**	**-37.37**	**-59.67**	**-25.63**	**-42.65**	**-69.65**	**-31.82**	**-50.74**

Table S22 Interaction energies (kCal·mol^{-1}) of ERβ with *GoldScore* poses of ligand 40

**Residue** #	**Type 1**			**Type 2**			**Type 3**			**Type 4**		
	**IE (CP)**	**IE (Raw)**	**IE (Avg)**	**IE (CP)**	**IE (Raw)**	**IE (Avg)**	**IE (CP)**	**IE (Raw)**	**IE (Avg)**	**IE (CP)**	**IE (Raw)**	**IE (Avg)**
295	-1.83	-0.23	-1.03	-1.54	-1.01	-1.28	-0.42	0.05	-0.19	-1.37	0.55	-0.41
298	-3.23	0.43	-1.40	-3.16	-1.86	-2.51	-2.56	3.99	0.72	-4.78	0.84	-1.97
299	-0.85	0.55	-0.15	-0.63	-0.52	-0.58	-4.26	-1.82	-3.04	-0.90	-0.52	-0.71
301	-1.45	-1.11	-1.28	-1.61	-1.20	-1.41	-1.94	-1.44	-1.69	-4.44	-1.87	-3.16
302	-1.15	-0.13	-0.64	-0.97	-0.06	-0.52	-1.13	0.38	-0.38	-2.29	2.03	-0.13
305	-28.25	-22.51	-25.38	-28.40	-22.47	-25.44	-27.05	-21.06	-24.06	-29.24	-22.89	-26.07
336	-2.73	0.03	-1.35	-3.05	-1.67	-2.36	-3.98	-2.37	-3.18	-1.13	-0.80	-0.97
339	-0.98	6.14	2.58	-0.58	5.80	2.61	-1.13	5.10	1.99	4.90	12.38	8.64
340	-3.75	-2.01	-2.88	-3.16	0.61	-1.28	-3.51	-2.36	-2.94	-1.42	0.94	-0.24
343	-1.88	0.06	-0.91	-2.81	0.82	-1.00	-2.24	-0.42	-1.33	-0.59	2.44	0.93
346	-8.06	-6.44	-7.25	-8.47	-6.61	-7.54	-7.51	-5.91	-6.71	-10.75	-8.79	-9.77
356	-3.10	-2.36	-2.73	-4.24	-2.97	-3.61	-3.44	-2.47	-2.96	-4.87	-3.12	-4.00
373	-0.20	-0.16	-0.18	-0.54	-0.34	-0.44	-0.68	-0.51	-0.60	-0.07	1.64	0.79
376	-0.46	-0.41	-0.44	-0.69	-0.49	-0.59	-0.85	-0.65	-0.75	-0.48	-0.45	-0.47
377	-0.22	-0.22	-0.22	-0.21	-0.21	-0.21	-0.24	-0.24	-0.24	-0.58	-0.44	-0.51
380	-0.35	-0.32	-0.34	-1.59	-0.63	-1.11	-0.25	-0.23	-0.24	-0.42	-0.34	-0.38
472	-4.12	-2.31	-3.22	-3.10	-0.73	-1.92	-0.30	2.28	0.99	-0.37	-0.37	-0.37
475	-3.19	-2.31	-2.75	-2.04	-0.19	-1.12	-3.09	-0.82	-1.96	-2.48	-1.71	-2.10
476	-4.08	0.45	-1.82	-3.56	0.70	-1.43	-4.53	-0.18	-2.36	-1.30	-0.75	-1.03
479	-0.65	-0.41	-0.53	-0.29	-0.23	-0.26	-0.15	-0.08	-0.12	0.23	0.28	0.26
491	-0.17	-0.16	-0.17	-0.08	-0.08	-0.08	-0.16	-0.16	-0.16	-0.15	-0.14	-0.15
**Total**	**-70.70**	**-33.43**	**-52.07**	**-70.72**	**-33.34**	**-52.03**	**-69.42**	**-28.92**	**-49.17**	**-62.50**	**-21.09**	**-41.80**

Table S23 Interaction energies (kCal·mol^{-1}) of ERβ with *GoldScore* poses of ligand 44

**Residue** #	**Type 1**			**Type 2**			**Type 3**			**Type 4**		
	**IE (CP)**	**IE (Raw)**	**IE (Avg)**	**IE (CP)**	**IE (Raw)**	**IE (Avg)**	**IE (CP)**	**IE (Raw)**	**IE (Avg)**	**IE (CP)**	**IE (Raw)**	**IE (Avg)**
295	-1.80	-0.16	-0.98	-1.69	-0.14	-0.92	-0.13	0.04	-0.05	-0.70	-0.16	-0.43
298	-3.44	0.13	-1.66	-4.14	-1.95	-3.05	-3.85	-2.80	-3.33	4.13	9.61	6.87
299	-1.05	0.19	-0.43	-1.24	-0.76	-1.00	-1.72	-1.36	-1.54	-0.44	-0.19	-0.32
301	-1.38	-1.06	-1.22	-1.87	-1.36	-1.62	-1.69	-1.20	-1.45	1.64	4.67	3.16
302	-1.10	-0.17	-0.64	-0.38	0.92	0.27	-1.39	-0.26	-0.83	8.40	13.27	10.84
305	-29.21	-23.51	-26.36	-27.94	-22.13	-25.04	-30.36	-24.16	-27.26	-28.61	-22.54	-25.58
336	-2.82	-0.31	-1.57	-2.13	-1.27	-1.70	-3.24	-2.06	-2.65	-2.22	-1.50	-1.86
339	-4.55	0.00	-2.28	-1.36	4.02	1.33	-0.65	6.28	2.82	-0.09	5.64	2.78
340	-3.61	-1.56	-2.59	0.67	3.26	1.97	-2.33	-0.18	-1.26	-0.76	2.00	0.62
343	-1.93	0.02	-0.96	1.96	5.43	3.70	-3.05	-1.04	-2.05	-1.92	0.78	-0.57
346	-7.83	-6.21	-7.02	-6.85	-5.23	-6.04	-8.15	-6.33	-7.24	-9.02	-7.00	-8.01
356	-3.10	-2.39	-2.75	-4.32	-2.29	-3.31	-3.72	-2.51	-3.12	-5.21	-3.68	-4.45
373	-0.22	-0.16	-0.19	-0.31	-0.17	-0.24	-1.50	0.38	-0.56	-1.68	-0.29	-0.99
376	-0.98	-0.68	-0.83	-0.38	-0.29	-0.34	-1.84	-0.95	-1.40	-0.89	-0.68	-0.79
377	-0.29	-0.29	-0.29	-0.25	-0.24	-0.25	-0.65	-0.44	-0.55	-0.42	-0.35	-0.39
380	-0.46	-0.42	-0.44	1.96	3.28	2.62	-0.62	-0.46	-0.54	-0.42	-0.35	-0.39
472	1.64	4.32	2.98	-3.10	-1.90	-2.50	-1.34	-1.00	-1.17	-0.76	-0.65	-0.71
475	-3.79	-2.81	-3.30	-5.23	-3.80	-4.52	-4.84	-1.96	-3.40	-4.28	-1.81	-3.05
476	-4.46	-0.52	-2.49	-3.63	0.75	-1.44	-0.49	2.32	0.92	-2.13	-0.33	-1.23
479	-0.64	-0.40	-0.52	-0.58	-0.27	-0.43	0.18	0.20	0.19	0.28	0.33	0.31
491	-0.13	-0.13	-0.13	-0.11	-0.10	-0.11	-0.54	-0.36	-0.45	-0.20	-0.20	-0.20
**Total**	**-71.15**	**-36.12**	**-53.64**	**-60.92**	**-24.24**	**-42.58**	**-71.92**	**-37.85**	**-54.89**	**-45.30**	**-3.43**	**-24.37**

Table S24 Interaction energies (kCal·mol^{-1}) of ERβ with *GoldScore* poses of ligand 57

**Residue #**	**Type 1**			**Type 2**			**Type 3**			**Type 4**		
	**IE (CP)**	**IE (Raw)**	**IE (Avg)**	**IE (CP)**	**IE (Raw)**	**IE (Avg)**	**IE (CP)**	**IE (Raw)**	**IE (Avg)**	**IE (CP)**	**IE (Raw)**	**IE (Avg)**
295	-1.98	0.32	-0.83	0.21	2.27	1.24	-0.15	0.07	-0.04	0.03	0.06	0.05
298	-8.90	-5.40	-7.15	-1.57	3.52	0.98	-4.29	-3.13	-3.71	12.96	18.80	15.88
299	-1.36	-0.88	-1.12	-3.86	-1.66	-2.76	-3.39	-2.25	-2.82	0.38	0.53	0.46
301	-1.42	-0.27	-0.85	-2.23	-1.66	-1.95	-1.59	-1.10	-1.35	-0.95	2.32	0.69
302	-0.84	-0.20	-0.52	0.33	2.03	1.18	-1.18	0.01	-0.59	-0.15	3.35	1.60
305	-33.83	-28.57	-31.20	-29.93	-23.64	-26.79	-33.22	-27.08	-30.15	-28.59	-23.12	-25.86
336	-0.98	-0.66	-0.82	-2.18	-1.34	-1.76	-3.12	-1.96	-2.54	-1.60	-1.08	-1.34
339	-4.05	-1.25	-2.65	-1.37	3.80	1.22	-0.16	6.46	3.15	0.73	6.10	3.42
340	-2.36	-0.27	-1.32	-2.96	-0.70	-1.83	0.68	5.12	2.90	-0.33	2.52	1.10
343	-3.07	-1.53	-2.30	4.87	8.67	6.77	-3.13	-1.10	-2.12	0.95	4.20	2.58
346	-5.14	-3.88	-4.51	-6.90	-5.32	-6.11	-4.69	-2.81	-3.75	-9.48	-7.62	-8.55
356	-4.60	-1.66	-3.13	-4.00	-2.02	-3.01	-3.93	-2.79	-3.36	-6.78	-3.88	-5.33
373	-0.02	2.08	1.03	-0.08	0.02	-0.03	-1.86	-0.13	-1.00	1.36	3.79	2.58
376	-1.32	0.71	-0.31	0.07	0.12	0.10	-1.65	-0.78	-1.22	-1.54	0.69	-0.43
377	-3.23	-2.03	-2.63	-0.06	-0.06	-0.06	-0.51	-0.36	-0.44	-2.20	-0.92	-1.56
380	-1.50	-1.14	-1.32	3.74	6.06	4.90	-0.71	-0.54	-0.63	-1.25	-0.65	-0.95
472	-0.91	-0.87	-0.89	-2.98	-2.08	-2.53	-1.18	-0.84	-1.01	-3.08	-2.34	-2.71
475	-7.08	-4.36	-5.72	-5.41	-4.28	-4.85	-4.60	-1.88	-3.24	-5.08	-2.09	-3.59
476	-3.12	-0.57	-1.85	-2.91	1.42	-0.75	-0.50	2.69	1.10	-0.28	0.47	0.10
479	-1.30	-0.81	-1.06	-0.40	-0.03	-0.22	0.13	0.15	0.14	0.44	0.44	0.44
491	-0.03	-0.02	-0.03	-0.19	-0.19	-0.19	-1.26	-0.72	-0.99	-0.23	-0.23	-0.23
**Total**	**-87.04**	**-51.26**	**-69.15**	**-57.81**	**-15.07**	**-36.44**	**-70.31**	**-32.97**	**-51.64**	**-44.69**	**1.34**	**-21.68**

Table S25 Interaction energies (kCal·mol^{-1}) of ERβ with *GoldScore* poses of ligand 62

**Residue #**	**Type 1**			**Type 2**			**Type 3**			**Type 4**		
	**IE (CP)**	**IE (Raw)**	**IE (Avg)**	**IE (CP)**	**IE (Raw)**	**IE (Avg)**	**IE (CP)**	**IE (Raw)**	**IE (Avg)**	**IE (CP)**	**IE (Raw)**	**IE (Avg)**
295	-2.09	-0.85	-1.47	0.05	3.51	1.78	-1.12	-0.71	-0.92	-0.79	0.36	-0.22
298	-5.93	-3.14	-4.54	1.79	7.46	4.63	-4.28	-0.37	-2.33	-4.07	1.40	-1.34
299	-3.34	-1.19	-2.27	-1.19	0.63	-0.28	-0.82	-0.25	-0.54	-0.97	-0.61	-0.79
301	-1.29	-0.54	-0.92	-1.25	1.39	0.07	-1.70	-1.20	-1.45	-2.17	-0.89	-1.53
302	-1.79	-0.97	-1.38	-3.39	-2.22	-2.81	-1.06	0.53	-0.27	0.28	2.77	1.53
305	-26.45	-22.19	-24.32	-6.62	-4.32	-5.47	-30.78	-24.66	-27.72	-35.22	-29.80	-32.51
336	-1.88	-1.33	-1.61	-2.88	-0.35	-1.62	-2.72	-0.64	-1.68	-2.09	-1.46	-1.78
339	-2.15	2.19	0.02	-5.36	-0.31	-2.84	0.73	8.39	4.56	-2.85	-1.79	-2.32
340	-3.22	0.85	-1.19	-0.49	2.77	1.14	-1.00	3.17	1.09	0.09	2.29	1.19
343	-4.03	-0.69	-2.36	-2.54	-0.03	-1.29	3.36	7.96	5.66	4.47	8.45	6.46
346	-7.47	-6.44	-6.96	-5.09	-4.19	-4.64	-8.55	-6.97	-7.76	-6.24	-4.53	-5.39
356	-4.93	-2.58	-3.76	-3.66	-1.66	-2.66	-3.45	-2.59	-3.02	-5.95	-1.90	-3.93
373	-0.72	0.39	-0.17	-0.73	-0.19	-0.46	-0.41	-0.23	-0.32	-1.21	-0.54	-0.88
376	-1.06	-0.60	-0.83	-0.66	-0.49	-0.58	-1.57	-0.95	-1.26	-1.14	-0.49	-0.82
377	-0.31	-0.15	-0.23	-0.22	-0.22	-0.22	-0.21	-0.21	-0.21	-0.46	-0.46	-0.46
380	-0.60	-0.39	-0.50	-0.25	-0.21	-0.23	-0.89	-0.81	-0.85	2.26	4.12	3.19
472	-0.74	-0.50	-0.62	-0.78	-0.38	-0.58	-0.60	3.19	1.30	6.84	11.30	9.07
475	-2.05	2.75	0.35	-10.65	-8.14	-9.40	-3.74	-1.70	-2.72	-6.64	-1.95	-4.30
476	-4.32	0.08	-2.12	-2.26	2.50	0.12	-4.37	-0.45	-2.41	-5.34	-2.24	-3.79
479	-0.90	-0.56	-0.73	-1.36	-0.75	-1.06	0.01	0.07	0.04	-0.30	-0.18	-0.24
491	-1.50	-0.94	-1.22	-0.31	-0.31	-0.31	-0.03	-0.03	-0.03	0.02	0.03	0.03
**Total**	**-76.77**	**-36.80**	**-56.79**	**-47.85**	**-5.51**	**-26.68**	**-63.20**	**-18.46**	**-40.83**	**-61.48**	**-16.12**	**-38.80**

Table S26 Interaction energies (kCal·mol^{-1}) of ERβ with *GoldScore* poses of ligand 68

Residue #	Type 1			Type 2			Type 3			Type 4		
	**IE (CP)**	**IE (Raw)**	**IE (Avg)**	**IE (CP)**	**IE (Raw)**	**IE (Avg)**	**IE (CP)**	**IE (Raw)**	**IE (Avg)**	**IE (CP)**	**IE (Raw)**	**IE (Avg)**
295	-1.63	-1.03	-1.33	1.20	4.96	3.08	-1.14	-0.55	-0.85	0.71	3.67	2.19
298	-5.68	-3.52	-4.60	10.39	16.09	13.24	-3.50	0.69	-1.41	-3.43	1.35	-1.04
299	-2.02	-0.49	-1.26	-2.14	-0.17	-1.16	-1.13	-0.41	-0.77	-1.60	-0.84	-1.22
301	-2.48	0.00	-1.24	2.47	5.15	3.81	-1.85	-1.32	-1.59	-0.89	-0.59	-0.74
302	-2.15	-0.95	-1.55	-2.32	-1.50	-1.91	-1.08	0.75	-0.17	-0.93	0.34	-0.30
305	-12.98	-6.51	-9.75	-2.46	-0.88	-1.67	-28.99	-22.91	-25.95	-11.76	-10.70	-11.23
336	-2.48	-1.76	-2.12	-0.60	2.53	0.97	-2.83	-0.75	-1.79	-1.94	-0.92	-1.43
339	-3.54	0.91	-1.32	0.63	6.41	3.52	0.73	8.08	4.41	-2.97	-1.16	-2.07
340	-2.14	-0.12	-1.13	-0.21	3.78	1.79	-4.72	-0.71	-2.72	2.35	6.68	4.52
343	-3.04	-1.07	-2.06	-2.18	0.71	-0.74	3.06	7.70	5.38	-3.46	-1.53	-2.50
346	-10.35	-8.90	-9.63	-6.71	-5.90	-6.31	-8.77	-7.20	-7.99	0.33	0.47	0.40
356	-4.53	-2.17	-3.35	-3.58	-0.80	-2.19	-3.47	-2.59	-3.03	-4.59	-2.99	-3.79
373	-1.45	0.11	-0.67	-0.71	-0.20	-0.46	-0.61	-0.44	-0.53	-1.34	0.79	-0.28
376	-1.29	-0.75	-1.02	-0.66	-0.49	-0.58	-1.89	-1.15	-1.52	-1.93	-0.97	-1.45
377	-0.49	-0.33	-0.41	-0.24	-0.24	-0.24	-0.28	-0.28	-0.28	-0.33	-0.33	-0.33
380	-0.82	-0.51	-0.67	-0.35	-0.31	-0.33	-0.56	-0.45	-0.51	-1.20	-0.03	-0.62
472	-0.48	-0.21	-0.35	-1.02	-0.49	-0.76	2.82	6.67	4.75	-0.63	-0.34	-0.49
475	-2.56	0.85	-0.86	-10.85	-8.30	-9.58	-2.68	-0.82	-1.75	-6.94	2.11	-2.42
476	-5.24	-1.72	-3.48	-3.73	0.88	-1.43	-5.42	-1.36	-3.39	0.60	6.73	3.67
479	-0.59	-0.53	-0.56	-1.50	-0.82	-1.16	-0.20	-0.12	-0.16	-1.40	0.65	-0.38
491	-0.50	0.02	-0.24	-0.23	-0.23	-0.23	-0.08	-0.08	-0.08	-0.10	-0.10	-0.10
**Total**	**-66.44**	**-28.68**	**-47.56**	**-24.80**	**20.18**	**-2.31**	**-62.59**	**-17.25**	**-39.92**	**-41.45**	**2.29**	**-19.58**

Table S27 Interaction energies (kCal·mol^{-1}) of ERβ with *GoldScore* poses of ligand 70

Residue #	Type 1			Type 2			Type 3			Type 4		
	**IE (CP)**	**IE (Raw)**	**IE (Avg)**	**IE (CP)**	**IE (Raw)**	**IE (Avg)**	**IE (CP)**	**IE (Raw)**	**IE (Avg)**	**IE (CP)**	**IE (Raw)**	**IE (Avg)**
295	-2.54	-0.81	-1.68	0.17	3.79	1.98	-0.81	-0.39	-0.60	-0.04	1.53	0.75
298	-6.63	-2.89	-4.76	-6.53	0.12	-3.21	-7.35	-4.77	-6.06	0.49	6.61	3.55
299	-1.97	0.65	-0.66	-1.25	0.88	-0.19	-0.98	-0.74	-0.86	-1.23	-0.76	-1.00
301	-0.17	2.90	1.37	1.97	5.65	3.81	-1.70	-0.53	-1.12	-2.26	-1.10	-1.68
302	-1.96	-1.18	-1.57	-2.25	-0.04	-1.15	-0.64	0.93	0.15	0.16	2.89	1.53
305	-14.68	-8.69	-11.69	-8.12	-5.06	-6.59	-34.28	-28.34	-31.31	-35.59	-29.98	-32.79
336	-2.24	-1.79	-2.02	-3.61	-1.49	-2.55	-2.48	-1.58	-2.03	-2.06	-1.00	-1.53
339	-3.69	-0.26	-1.98	-9.34	-4.96	-7.15	-2.60	2.34	-0.13	-4.60	-2.52	-3.56
340	-2.07	-0.43	-1.25	-1.59	0.76	-0.42	-5.02	-1.88	-3.45	5.71	9.68	7.70
343	-3.53	-1.63	-2.58	-2.75	-0.57	-1.66	-3.89	-1.76	-2.83	-1.11	1.09	-0.01
346	-8.50	-6.99	-7.75	-5.96	-4.93	-5.45	-8.02	-6.95	-7.49	-5.75	-4.54	-5.15
356	-4.57	-1.90	-3.24	-5.33	-2.39	-3.86	-4.74	-3.40	-4.07	-5.89	-3.76	-4.83
373	-0.89	0.08	-0.41	-0.68	-0.22	-0.45	-0.90	1.84	0.47	-1.29	-0.66	-0.98
376	-0.78	-0.52	-0.65	-0.54	-0.41	-0.48	-2.63	-0.18	-1.41	-0.97	-0.70	-0.84
377	-0.36	-0.23	-0.30	-0.22	-0.22	-0.22	-2.95	-1.49	-2.22	-0.46	-0.46	-0.46
380	-0.53	-0.34	-0.44	-0.23	-0.20	-0.22	-1.08	-0.76	-0.92	-1.89	-1.08	-1.49
472	-0.31	-0.24	-0.28	-0.78	-0.42	-0.60	-1.78	-1.22	-1.50	17.39	22.10	19.75
475	-6.16	-3.75	-4.96	-9.88	-7.56	-8.72	0.18	5.13	2.66	-4.60	0.70	-1.95
476	-3.35	1.09	-1.13	-2.87	1.87	-0.50	-2.77	0.75	-1.01	-6.83	-2.93	-4.88
479	-0.95	-0.66	-0.81	-1.44	-0.81	-1.13	0.26	0.36	0.31	-0.60	-0.42	-0.51
491	-1.55	-0.74	-1.15	-0.23	-0.23	-0.23	-0.15	-0.13	-0.14	0.01	0.01	0.01
**Total**	**-67.43**	**-28.33**	**-47.88**	**-61.46**	**-16.44**	**-38.95**	**-84.33**	**-42.77**	**-63.55**	**-51.41**	**-5.30**	**-28.36**

Table S28 Comparison of ranking based on the experimental IC_{50} values, the average IE from KEM-CP and fitting score of the best poses of the ERβ ligands.

	Best Poses (from all Types)					
**Ligand**	**Expt IC_{50}**		**KEM-CP**		**GoldScore**	
#	**Value (nM)**	**Ranking**	**IE (kCal·mol^{-1})**	**Ranking**	**Fitness Score**	**Ranking**
15	2.52±1.3	**9**	**-41.90**	**10**	57.1	**1***
25	2.8±0.1	**10**	**-64.69**	**2***	51.87	**10**
27	2.3±0.1	**7**	**-56.42**	**6**	53.05	**9**
29	1.4±0.6	**4**	**-58.45**	**4**	55.15	**6**
40	1.6±0.7	**5**	**-52.07**	**8**	53.38	**8**
44	2.3±1.7	**8**	**-54.89**	**7**	55.86	**3***
57	0.5±0.5	**1**	**-69.15**	**1**	55.29	**4**
62	2.1±0.9	**6**	**-56.79**	**5**	55.28	**5**
68	1.2±0.7	**3**	**-47.56**	**9***	56.13	**2**
70	1.1±1.6	**2**	**-63.55**	**3**	54.13	**7**
**Correlation**				***0.88***		***0.71***

** Ligands with highest disagreements (difference of 5 to 8) in ranking were excluded for the calculation of the correlation coefficient.*

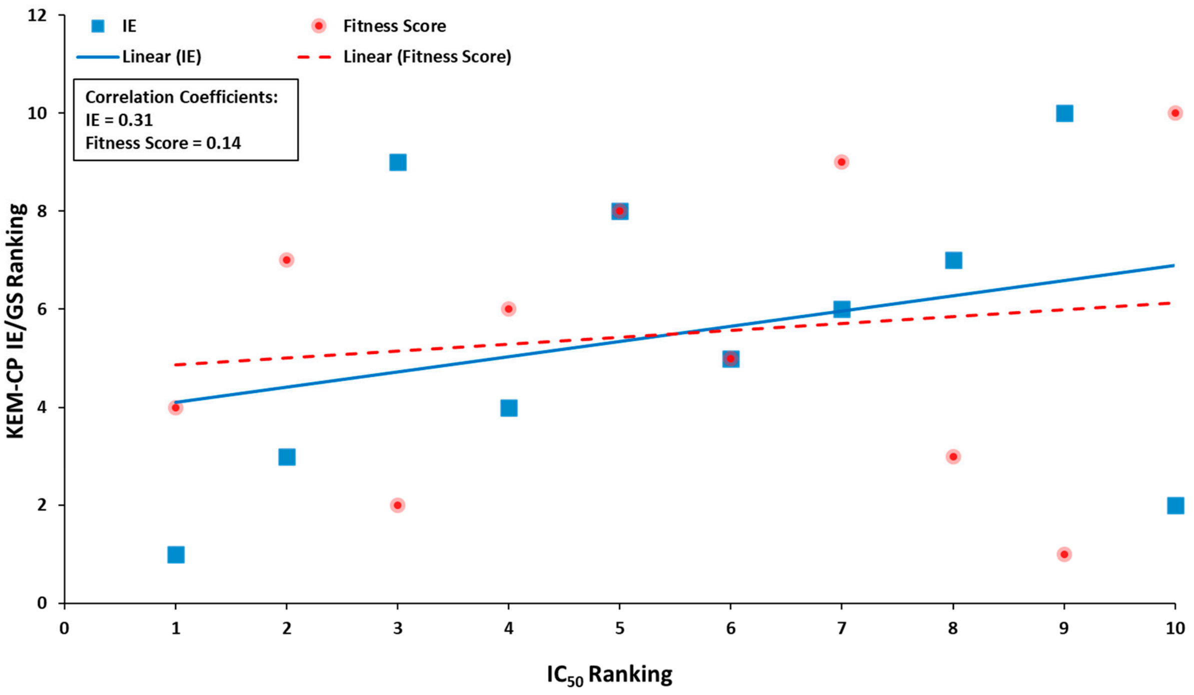

Figure S5 Plot showing correlations of rankings based on the average KEM-CP IE and *GoldScore* fitness score with the ranking based on IC_{50} values for best poses of all 10 ligands (data from **Table S28**).

Table S29 Comparison of ranking based on the experimental IC_{50} values, the average IE from KEM-CP and fitting score of the pose Type 1 of the ERβ ligands.

Pose Type 1						
	Expt IC_{50}		KEM-CP		GoldScore	
**Ligand**	**Value (nM)**	**Ranking**	**IE (kCal·mol^{-1})**	**Ranking**	**Fitness Score**	**Ranking**
15	2.52±1.3	**9**	-40.33	**10**	57.10	**1***
25	2.8±0.1	**10**	**-64.69**	**2***	50.76	**9**
27	2.3±0.1	**7**	-49.90	**7**	49.68	**10**
29	1.4±0.6	**4**	**-58.45**	**3**	52.28	**8**
40	1.6±0.7	**5**	**-52.07**	**6**	53.38	**7**
44	2.3±1.7	**8**	-53.64	**5**	55.86	**3***
57	0.5±0.5	**1**	**-69.15**	**1**	55.29	**4**
62	2.1±0.9	**6**	**-56.79**	**4**	55.28	**5**
68	1.2±0.7	**3**	**-47.56**	**9***	56.13	**2**
70	1.1±1.6	**2**	-47.88	**8**	53.93	**6**
**Correlation**				***0.55***		***0.67***

** Ligands with highest disagreements (difference of 5 to 8) in ranking were excluded for the calculation of the correlation coefficient.*

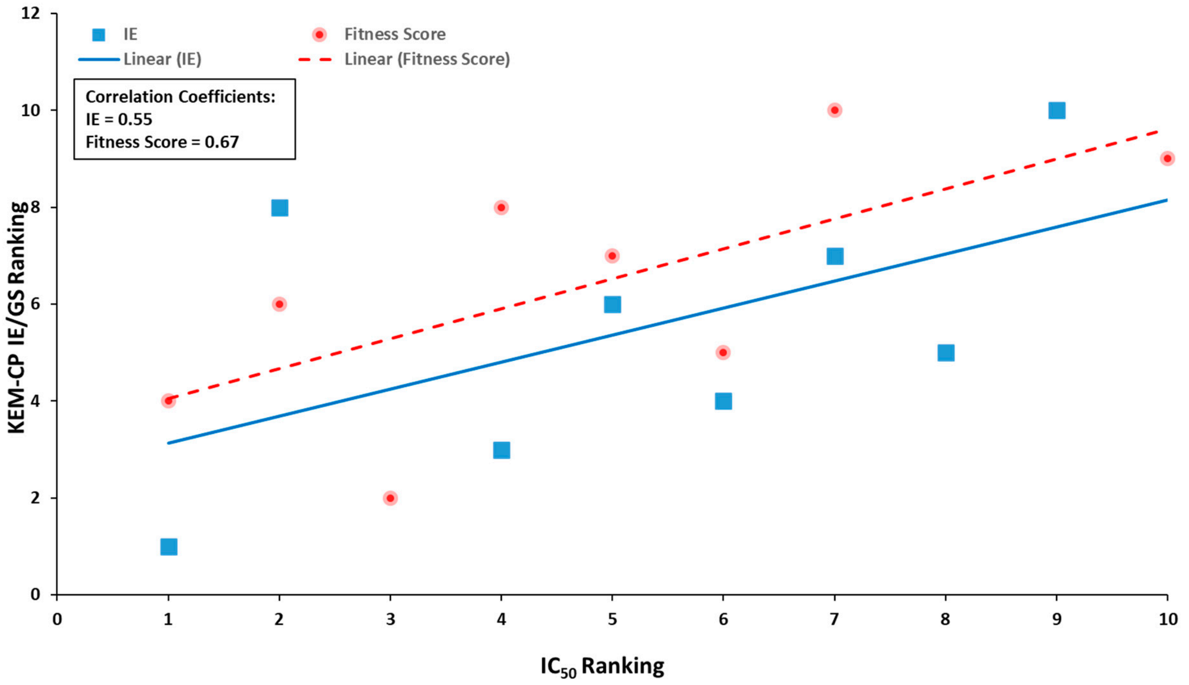

Figure S6 Plot showing correlations of rankings based on the average KEM-CP IE and *GoldScore* fitness score with the ranking based on IC_{50} values for pose Type 1 (data from **Table S34**)

Table S30 Comparison of ranking based on the experimental IC_{50} values, the average IE from KEM-CP and fitting score of the pose Type 3 of the ERβ ligands.

**Pose Type 3**						
	**Expt IC_{50}**		**KEM-CP**		**GoldScore**	
**Ligand**	**Value (nM)**	**Ranking**	**IE ($kCal \cdot mol^{-1}$)**	**Ranking**	**Fitness Score**	**Ranking**
15	2.52±1.3	**9**	**-41.90**	**8**	55.11	**3***
25	2.8±0.1	**10**	-57.96	**2***	50.73	**9**
27	2.3±0.1	**7**	-43.79	**6**	50.02	**10**
29	1.4±0.6	**4**	-42.65	**7**	51.99	**8**
40	1.6±0.7	**5**	-49.17	**5**	52.11	**7**
44	2.3±1.7	**8**	**-54.89**	**3**	54.53	**4**
57	0.5±0.5	**1**	-51.64	**4**	52.72	**6***
62	2.1±0.9	**6**	-40.83	**9**	55.12	**2**
68	1.2±0.7	**3**	-39.92	**10***	55.18	**1**
70	1.1±1.6	**2**	**-63.55**	**1**	54.13	**5**
**Correlation**				***0.48***		***0.41***

** Ligands with highest disagreements (difference of 5 to 8) in ranking were excluded for the calculation of the correlation coefficient.*

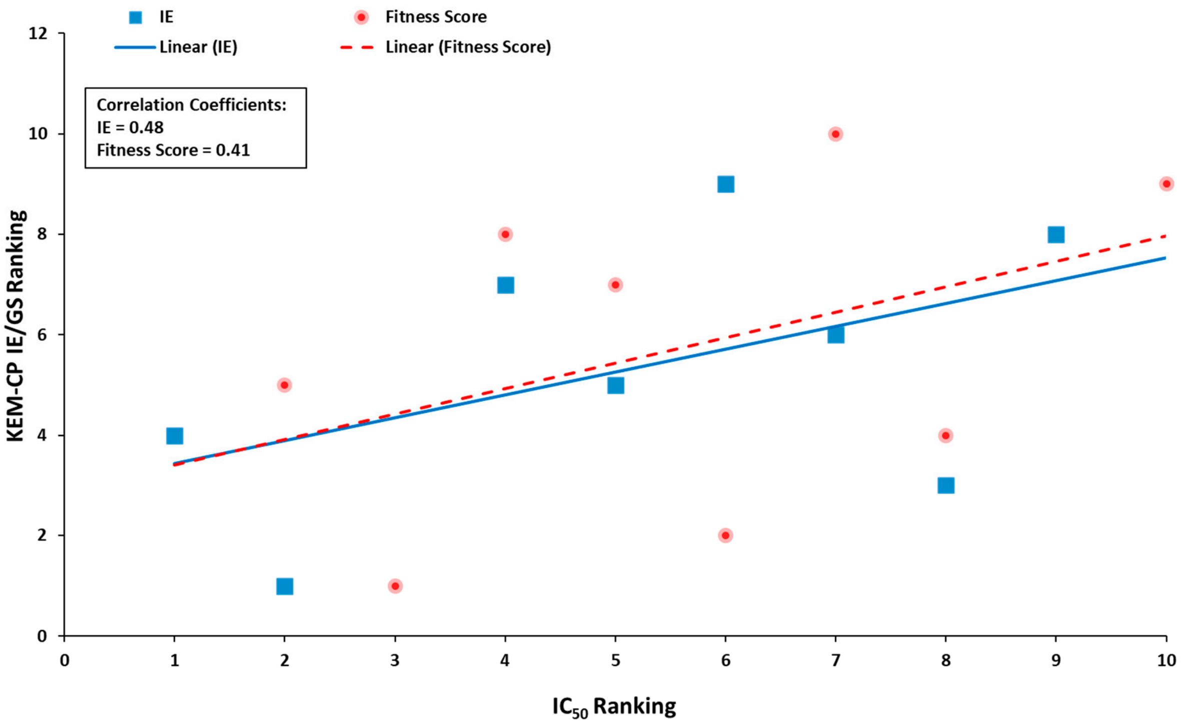

Figure S7 Plot showing correlations of rankings based on the average KEM-CP IE and *GoldScore* fitness score with the ranking based on IC_{50} values for pose Type 3 (data from **Table S35**)

1.1 Benchmarking of scoring functions

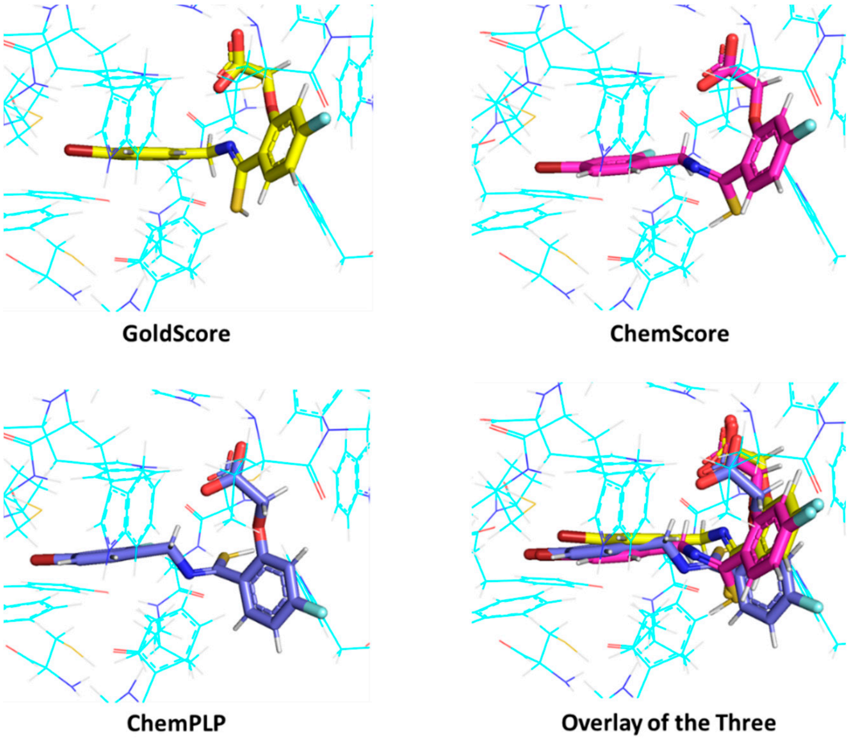

Figure S8 Best poses of IDD594 in hAR using different scoring functions

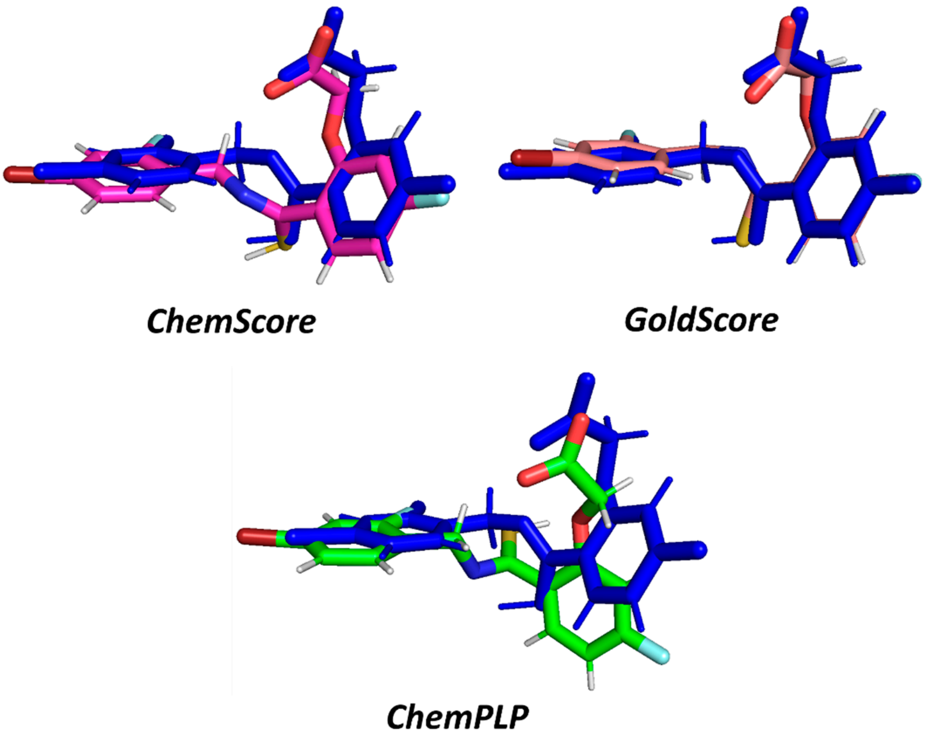

Figure S9 Comparison of IDD594 poses generated by different scoring functions with respect to the crystal geometry (blue)

Table S31 Interaction energies (kCal·mol^{-1}) of hAR-IDD594 complex

**IDD594 Crystal Geometry**			
**Residue #**	**CP corrected**	**Raw**	**Avg**
20	-2.29	-7.16	-4.73
47	-0.38	-4.13	-2.26
48	-5.34	-17.36	-11.35
79	-4.05	-5.35	-4.70
80	-0.85	-1.28	-1.07
110	-23.07	-28.29	-25.68
111	-26.93	-37.37	-32.15
113	2.75	-0.74	1.01
115	-1.68	-3.89	-2.79
122	-3.06	-5.81	-4.44
219	-2.31	-4.03	-3.17
298	-0.88	-2.06	-1.47
299	0.45	-2.05	-0.80
300	-2.27	-8.06	-5.17
303	-1.16	-3.42	-2.29
309	-1.61	-3.13	-2.37
310	-1.33	-1.45	-1.39

**Total**	**-74.01**	**-135.58**	**-104.80**

Table S32 Interaction energies (kCal·mol^{-1}) of hAR with *GoldScore* pose of ligand 19

	GoldScore		
**Residue #**	**CP corrected**	**Raw**	**Avg**
20	-1.88	-8.22	-5.05
47	-2.38	-3.81	-3.10
48	-24.81	-32.68	-28.75
79	-4.40	-5.99	-5.20
80	-0.69	-1.26	-0.98
110	-20.41	-25.60	-23.01
111	-23.37	-36.44	-29.91
113	1.15	-1.64	-0.25
115	-1.95	-3.67	-2.81
122	-3.78	-5.66	-4.72
219	-4.38	-6.96	-5.67
298	0.08	-2.41	-1.17
299	1.77	-2.34	-0.29
300	-2.53	-8.27	-5.40
303	-1.22	-2.20	-1.71
309	-1.56	-2.49	-2.03
310	-1.37	-1.47	-1.42
**Total**	**-91.73**	**-151.11**	**-121.42**

Table S33 Interaction energies (kCal·mol^{-1}) of hAR with *ChemScore* pose of ligand 19

	ChemScore		
**Residue #**	**CP corrected**	**Raw**	**Avg**
20	-3.64	-6.68	-5.16
47	-2.27	-4.52	-3.40
48	-24.3	-31.28	-27.79
79	-5.24	-6.92	-6.08
80	-0.98	-1.12	-1.05
110	-19.72	-24.28	-22.00
111	-27.51	-37.07	-32.29
113	7.01	2.19	4.60
115	0.26	-1.43	-0.59
122	-1.46	-5.93	-3.70
219	-1.32	-3.55	-2.44
298	-0.47	-1.44	-0.96
299	0.29	-1.49	-0.60
300	1.48	-5.74	-2.13

303	-0.08	-4.1	-2.09
309	1.61	-3.23	-0.81
310	-1.59	-2.42	-2.01
**Total**	**-77.93**	**-139.01**	**-108.47**

Table S34 Interaction energies (kCal·mol^{-1}) of hAR with *ChemPLP* pose of ligand 19

	ChemPLP		
**Residue #**	**CP corrected**	**Raw**	**Avg**
20	-1.92	-3.72	-2.82
47	-1.94	-2.24	-2.09
48	-19.53	-23.98	-21.76
79	-5.11	-8.06	-6.59
80	-1.2	-1.56	-1.38
110	-16.8	-20.29	-18.55
111	-26.49	-35.27	-30.88
113	17.62	10.35	13.99
115	4.22	1.65	2.94
122	-4.36	-6.78	-5.57
219	8.01	3.11	5.56
298	-1.71	-2.62	-2.17
299	1.5	-0.01	0.75
300	-2.13	-8.67	-5.40
303	-0.97	-4.75	-2.86
309	7.52	1.15	4.34
310	-1.47	-2.99	-2.23
**Total**	**-44.76**	**-104.68**	**-74.72**

Table S35 Interaction energy (IE) of hAR with IDD594 poses generated by different scoring function and with the crystal geometry of IDD594 and RMSD of the docked poses with respect to the crystal geometry.

**Source of Pose**	**IE (CP Corrected) (kCal·mol^{-1})**	**IE (Raw) (kCal·mol^{-1})**	**Average IE (kCal·mol^{-1})**	**RMSD (Å)**
**Crystal geometry**	-74.01	-135.58	-104.80	0.000
GoldScore	-91.73	-151.11	-121.42	0.137
ChemScore	-77.93	-139.01	-108.47	0.506
ChemPLP	-44.76	-104.68	-74.72	0.984

The pairwise IE for the crystal geometry IDD594 and the docked poses are listed in **Table S1-S4.** For *ChemPLP*, the best pose (rank 1) provided the higher average IE (~ -

75 kCal·mol^{-1}) with RMSD of ~1 Å (highest). For *ChemScore,* the best pose with reasonable RMSD (~0.5 Å) give rise to an average IE (~ -108 kCal·mol^{-1}) comparable to that of the crystal geometry. However, *GoldScore* resulted the most negative average IE (~ -121 kCal·mol^{-1}) and least RMSD. Thus, a strong correlation was observed between the average IE and the RMSD.

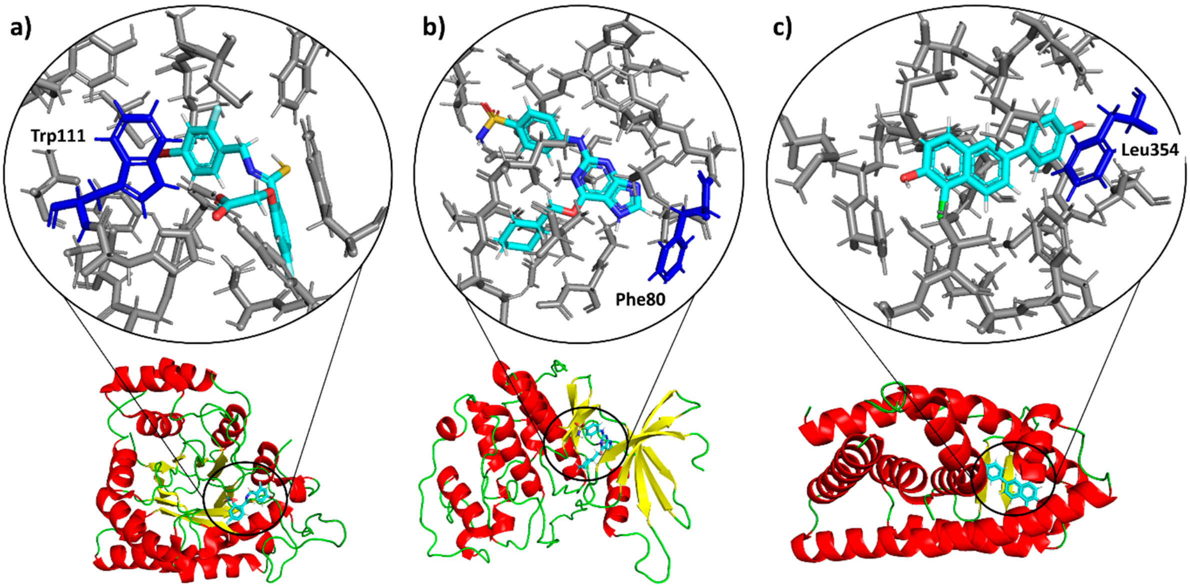

Figure S10 Views of the active sites of **(a)** hAR-IDD594, **(b)** CDK2-NU6102 and **(c)** ERB-4NA highlighting the complexed ligand (cyan) and the residues chosen as the active site centre (blue).

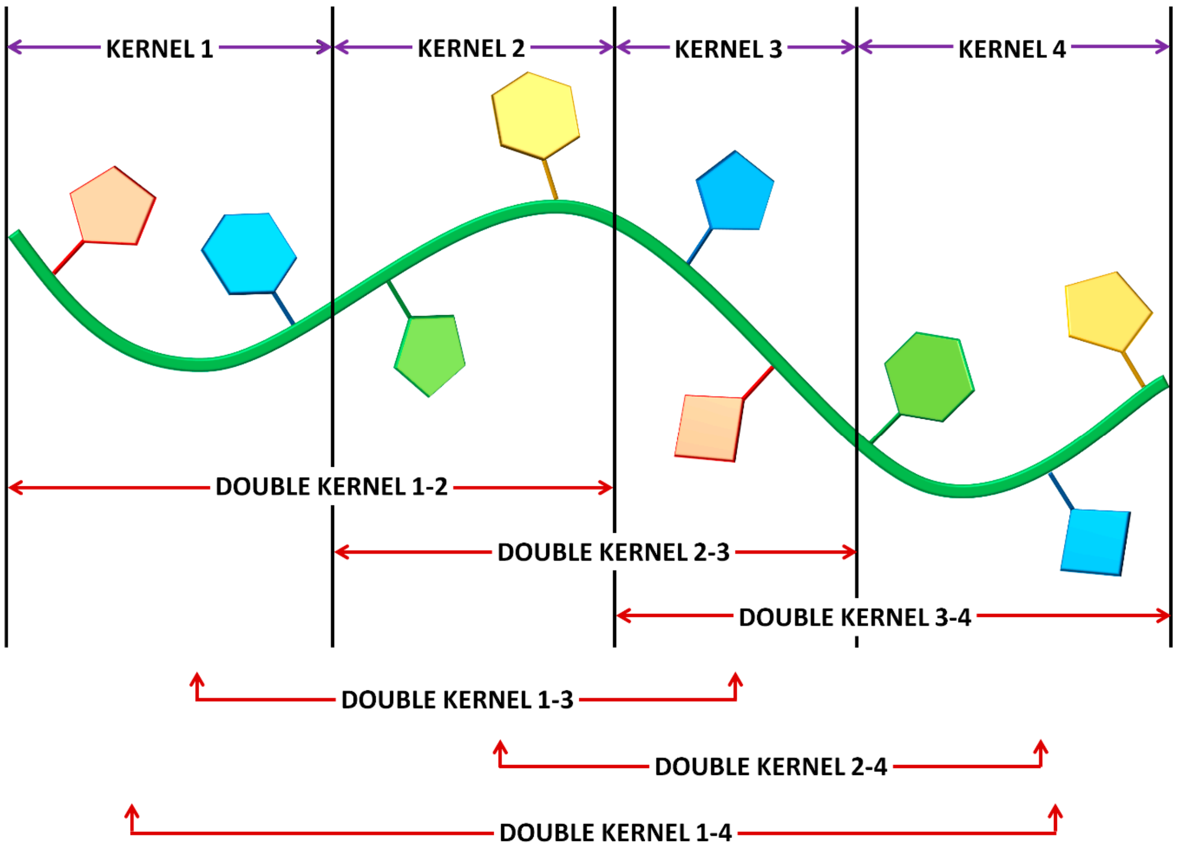

Figure S11 Representation of the fragmentation of macromolecule as single and double kernels

Reference

1. Ferrari, A.M.; Degliesposti, G.; Sgobba, M.; Rastelli, G. Validation of an automated procedure for the prediction of relative free energies of binding on a set of aldose reductase inhibitors. *Bioorg. Med. Chem.* **2007**, *15*, 7865–7877, doi:10.1016/j.bmc.2007.08.019.

2. Hardcastle, I.R.; Arris, C.E.; Bentley, J.; Boyle, F.T.; Chen, Y.; Curtin, N.J.; Endicott, J.A.; Gibson, A.E.; Golding, B.T.; Griffin, R.J.; et al. N 2 -Substituted O 6 -Cyclohexylmethylguanine Derivatives: Potent Inhibitors of Cyclin-Dependent Kinases 1 and 2. *J. Med. Chem.* **2004**, *47*, 3710–3722, doi:10.1021/jm0311442.

3. Mewshaw, R.E.; Edsall, R.J.; Yang, C.; Manas, E.S.; Xu, Z.B.; Henderson, R.A.; Keith, J.C.; Harris, H.A. ERβ Ligands. 3. Exploiting Two Binding Orientations of the 2-Phenylnaphthalene Scaffold To Achieve ERβ Selectivity. *J. Med. Chem.* **2005**, *48*, 3953–3979, doi:10.1021/jm058173s.
